# A drought stress‐induced MYB transcription factor regulates pavement cell shape in leaves of European aspen (*Populus tremula*)

**DOI:** 10.1111/nph.71399

**Published:** 2026-07-05

**Authors:** Sijia Liu, Siamsa M. Doyle, Kathryn M. Robinson, Zahra Rahneshan, Nathaniel R. Street, Stéphanie Robert

**Affiliations:** ^1^ Umeå Plant Science Centre, Department of Forest Genetics and Plant Physiology Swedish University of Agricultural Sciences Umeå SE‐901 83 Sweden; ^2^ Department of Grassland Science, College of Horticulture Shenyang Agricultural University Shenyang 110866 China; ^3^ Umeå Plant Science Centre, Department of Plant Physiology Umeå University Umeå SE‐901 87 Sweden; ^4^ SciLifeLab Umeå University Umeå SE‐901 87 Sweden

**Keywords:** cell shape, drought, European aspen (*Populus tremula*), GWAS, leaf pavement cells

## Abstract

- Leaf pavement cells of many plant species develop jigsaw puzzle‐like shapes in which neighboring cells interdigitate, providing an ideal model for the study of cell shape regulation. Such shapes are likely to reduce tissue‐wide mechanical stress, which is influenced by environmental conditions, such as drought stress.
- We analyzed pavement cell shape complexity in a natural population of European aspen (*Populus tremula*) genotypes and used a genome‐wide association study (GWAS) to identify a candidate gene in cell shape regulation, Potra2n8c18226, encoding the transcription factor MYB305a. We subsequently validated a role for *MYB305a* in regulating aspen leaf pavement cell shape.
- We then demonstrated that drought stress strongly induces *MYB305a* promoter expression in these cells and provided evidence that *MYB305a* plays a role in regulating pavement cell shape in response to drought. Finally, we observed correlations of pavement cell shape complexity with water‐use efficiency and average precipitation at the original sampling sites, in the natural aspen population.
- Taken together, our results suggest climatic variables affect shape complexity of pavement cells in aspen leaf and provide a foundation for future mechanistic studies on this process, by implicating the involvement of the transcription factor MY305a.

## Graphical abstract

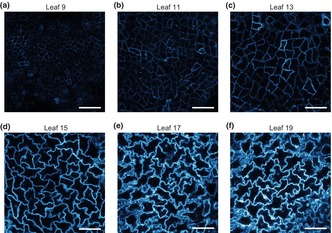

## Introduction

The cell wall ultimately defines the shape of plant cells. Cell shape is highly varied across tissues and is adapted to achieve the specialized functions of the cell. In many plant species, the pavement cells of the leaf epidermis develop an intriguing jigsaw puzzle‐like shape in which neighboring cells interdigitate with each other in a coordinated manner. Studies of pavement cells, primarily in Arabidopsis (*Arabidopsis thaliana*), have led to insights into how the cell wall defines the cell shape, as well as some of the hormonal and molecular signaling pathways involved (for details, see the following suggested reviews: Eng & Sampathkumar, 2018; Lin & Yang, 2020; Liu *et al*., 2021; Zuch *et al*., 2022; van Spoordonk *et al*., 2023). Several functional roles for the puzzle shape of pavement cells have been hypothesized over the years, including cell spacing, increased cell–cell contacts and adhesion, improved resistance to tension, and enhanced mechanical strength (Liu *et al*., 2021). However, pavement cell interdigitation is now thought to primarily function in alleviating the mechanical stress predicted to be generated in the epidermis of broad leaves during their near‐isotropic expansion, a hypothesis that has been supported by substantial computational modeling efforts (Sapala *et al*., 2018; Bidhendi *et al*., 2019; Trozzi *et al*., 2026). Such isotropic cell expansion results in enhanced turgor pressure‐induced mechanical stress in the cell walls of the epidermis, which are dispersed by the formation of cell outgrowths, reducing maximal wall stress at the middle of the cell and decreasing the risk of cell rupture or outward bulging at the leaf surface (Sapala *et al*., 2018). Since environmental conditions such as water availability can influence cellular turgor pressure in the leaf, and turgor pressure determines mechanical stress patterns in the epidermal cell walls, plasticity of pavement cell shape during leaf development may facilitate adaptation to changing local environmental conditions. Interdigitated pavement cells can therefore be thought of as model cells for improving our understanding of how plants coordinate cell morphology in a tissue context and in response to the environment.

Although *Populus* species also display jigsaw puzzle‐shaped leaf pavement cells (Liu *et al*., 2021), there has been little work done to investigate epidermal cell shape regulation in the *Populus* genus. These species are models for forest tree research due to their relatively small genome size, genetic diversity, amenability to transformation, ease of vegetative propagation, fast growth rate, usefulness for industrial production of paper materials, and potential for bioenergy production (Nilsson *et al*., 1992; Bryant *et al*., 2020; Zhang *et al*., 2023). A fundamental understanding of growth and development in *Populus* is therefore important for the continued progression of biotechnological advances in these woody perennial species. Furthermore, understanding how tree growth and development respond to environmental challenges is increasingly important in the context of future climate change.

On both the adaxial (upper) and abaxial (lower) sides of Arabidopsis leaves, the multiple asymmetrical divisions of small, undifferentiated meristemoid cells in stomatal cell lineage complexes are a major source of pavement cell origin (Geisler *et al*., 2000; Liu *et al*., 2021). The resulting young pavement cells subsequently develop outgrowths and interdigitate with their neighbors as they expand. By contrast, only the abaxial leaf side of European aspen (*Populus tremula*) develops stomata, and the pavement cells on both leaf sides seem to arise from the rather symmetrical divisions of pre‐existing interdigitated cells, resulting in straight cell walls that subsequently undulate as the sister cells continue to grow (Liu *et al*., 2021). These rather dissimilar processes of cell development suggest that pavement cell shape regulatory mechanisms may be different between Arabidopsis and aspen. Furthermore, high diversity of pavement cell shape with shallow phylogenetic signal (low similarity among closely related species) was found in the eudicots (Vőfély *et al*., 2019), supporting the idea that eudicot species may have evolved different regulatory mechanisms for this process.

This inspired us to seek regulators of pavement cell shape in aspen leaves. We analyzed leaf pavement cell shape complexity and size features in a natural population of European aspen genotypes, revealing variation in cell shape and size across the population. We then used a genome‐wide association study (GWAS) approach to identify a candidate gene, encoding a MYB family transcription factor, in aspen pavement cell shape regulation. We subsequently validated such a role for the GWAS candidate, *MYB305a* (Potra2n8c18226), through transgenic approaches. Since many MYB transcription factors play roles in drought stress responses (Baldoni *et al*., 2015), we next applied drought treatments to our trees. We demonstrated that *MYB305a* promoter expression is strongly drought‐induced in aspen leaf epidermis and found evidence supporting the involvement of *MYB305a* in regulating pavement cell shape responses to drought stress in this species.

## Materials and Methods

### Plant material and growth conditions

For phenotyping of pavement cells from adult trees in the field, mature preformed leaves (leaves that emerge from buds each Spring) were harvested from genotypes of the Swedish Aspen (SwAsp) collection of European aspen (*Populus tremula* L.) maintained by Skogforsk in the common garden at Sävar, Northern Sweden. Only mature, undamaged, nonshaded leaves were selected, and they were harvested from a height of *c*. 1.5 m above the ground. The first and last leaves from leaf cohorts originating from the same bud were avoided. Two to three leaves from each of one to five trees, from each of 106 different genotypes, were collected in plastic bags and stored at 4°C for up to several days. The total number of leaves analyzed was 685, from a total of 234 trees.

For all further experiments, trees of SwAsp genotypes and hybrid aspen (*Populus tremula* L. × *tremuloides* Michx.) clone T89 were obtained from the *in vitro* collections of *Populus* species maintained via shoot cutting propagation by the Umeå Plant Science Centre (UPSC) Poplar Transgenics Facility. For details on *in vitro* plant growth, see Supporting Information Methods S1. For growth in soil, 3‐wk‐old *in vitro* plants were potted and immediately covered with plastic bags, which were removed 2 wk later. For experiments with SwAsp genotypes or transgenic lines, the plants were then grown in a controlled glasshouse, or in an automated conveyor network (WIWAM Conveyor, SMO, Eeklo, Belgium) in a controlled growth room at the UPSC Tree Phenotyping Platform, respectively. The trees were grown under an 18 h : 6 h, 22°C : 18°C, day : night regime with 200 μmol m^−2^ s^−1^ light intensity and 60% relative humidity. In the platform, both white (FL300 Sunlight v.1.1) and red (custom‐made FL100 725–735 nm) LED lamps (Senmatic A/S, Søndersø, Denmark) were used and all side shoots were removed from the trees 3 and 6 wk after potting. Excluding the drought experiments, the trees were regularly fertilized with Rika S (SW Horto AB, Hammenhög, Sweden) and leaves were harvested 6 or 8 wk after potting. The trees at the platform were photographed and measured for shoot height 8 wk after potting. For the drought experiments, trees were grown in the platform for 5 wk, during which time the pots were watered daily to a level of 1.7 kg water per kg soil (soil target humidity (TH) 1.7). Drought conditions were then initiated by watering less each day for 10 d, reducing the TH by 0.1 daily, and reaching a final level of TH 0.7, at which point leaves were harvested. At the same time the drought was initiated, the control, nondrought trees were watered more, increasing the TH by 0.1 daily and then maintained at TH 1.9 until leaf harvesting.

### Microscopy imaging and phenotyping of pavement cells

All microscopic observations were made on samples cut from the middle of the blade on one side of the mid‐vein. Epidermal cells on the adaxial leaf side were imaged at three random positions per leaf sample, avoiding those cells overlying the veins. For analysis of pavement cell shape in preformed leaves, the leaf samples were fixed by incubation in 4% paraformaldehyde in 25 mM phosphate‐buffered saline (PBS; 8 g l^−1^ NaCl, 0.2 g l^−1^ KCl, 1.78 g l^−1^ Na_2_HPO_4_·2H_2_O, 0.24 g l^−1^ KH_2_PO_4_, pH 7.4) under a vacuum for 1 h, followed by storage overnight at 4°C. The samples were then washed and dehydrated according to the following protocol: 25 mM PBS for 10 min three times, 30% ethanol for 10 min three times, 50% ethanol for 10 min three times, and then stored in 50% ethanol at 4°C. Before microscopy imaging, the leaf samples were rehydrated by incubating for 1 h in each of the following ethanol series: 40, 30, 20, and 10%, before transferring to PBS. The samples were subsequently stained in 0.1% calcofluor white for 30 min, followed by three rinses with water, before transferring to PBS overnight. Samples were mounted in PBS and Z‐stack imaging encompassing all visible calcofluor white fluorescence, with 2 μm distance between each slice, was performed on an LSM 780 confocal laser scanning microscope (Carl Zeiss Microscopy, Oberkochen, Germany). The ImageJ (Fiji) macro SurfCut (Erguvan *et al*., 2019) was used to extract a layer of signal relative to the sample surface in each Z‐stack, followed by application of the ImageJ Gaussian blur tool to reduce the background noise. In those images with sufficient quality, the ImageJ plugin PaCeQuant (Möller *et al*., 2017) from the toolbox MiToBo (Möller *et al*., 2016) was then applied for automated segmentation of the pavement cell outlines and analysis of circularity (measure of deviation from a perfect circle), branch count (number of branches in the cell skeleton), lobe number (number of cell outgrowths), solidity (ratio of cell area to area of the cell's convex hull, a convex polygon fitting around the cell), nonlobe area (cell area excluding lobes), total cell area, perimeter, length and width (lengths of the major and minor axes, respectively, of an ellipse fitting around the cell), and convex hull perimeter. Lobeyness was then calculated as the convex hull perimeter divided by the cell perimeter (Sapala *et al*., 2018). In those images without sufficient quality for automated segmentation, the cell outlines were drawn by hand in Photoshop (Adobe, San Jose, CA, USA) from maximum projections of the Z‐stacks, before extraction and measurements of cell shape and size features with PaCeQuant.

For analysis of pavement cell shape in terminal leaves (except for *pMYB305a::green fluorescent protein; GFP*), which are formed at the terminal meristem and emerge directly from the stem, the leaf samples were cut on the day of leaf harvesting and stored in 70% ethanol at 4°C. The samples were rehydrated and cleared immediately before imaging, according to the following protocol: 50% ethanol for 15 min, 30% ethanol for 15 min, 10% ethanol for 15 min, PBS for 15 min, 5% NaOCl overnight, PBS for 15 min, and fresh PBS. Imaging was performed on a DMi8 epifluorescence microscope (Leica Microsystems, Wetzlar, Germany) equipped with a camera. For imaging *pMYB305a::GFP* leaves, each sample was cut from freshly harvested leaves immediately before mounting in PBS and GFP fluorescence was imaged on an LSM 880 confocal laser scanning microscope (Carl Zeiss Microscopy). For all terminal leaf microscopy images, the cell outlines were drawn by hand, extracted, and the cell shape features and area measured as described above.

All raw pavement cell shape and size feature data are publicly available at https://doi.org/10.5061/dryad.kwh70rzm9.

### Genome‐wide association study

Median values per leaf sample were calculated for each cell shape and size feature to obtain one value per phenotype and biological replicate. Data for each phenotype were then parsed through a pipeline for data quality control and the calculation of a best linear unbiased predictor (BLUP) of the random effects that was subsequently used in the GWAS, as described previously (Robinson *et al*., 2024). The GWAS and downstream analyses of the results were performed as detailed previously (Robinson *et al*., 2024). Briefly, for each of the shape and size features, phenotypic BLUP values for each European aspen genotype and 6806 717 single‐nucleotide polymorphisms (SNPs) were analyzed using linear mixed models in GEMMA 0.98.1 (Zhou & Stephens, 2012) with two covariates: a genetic relatedness matrix and the latitude of origin of the SwAsp genotype to avoid confounding growth effects (Wang *et al*., 2018; Robinson *et al*., 2024). SNPs with minor allele frequency (MAF) < 0.05, Hardy–Weinberg equilibrium *P‐*value < 10e‐6, and missingness > 0.1 were removed before the analyses. The GWAS was conducted using 92 unrelated European aspen genotypes for which both phenotypic data and SNP data were available. Data were prepared and plotted in the R statistical computing environment (R Core Team, 2024), using the qqman package for manhattan and QQ plots (Turner, 2018) and the ggplot2 package for allele boxplots (Wickham, 2016). Broad‐sense heritability was estimated using the heritability package in R (Kruijer *et al*., 2015). Genetic correlations were estimated as described previously (Mähler *et al*., 2020; Robinson *et al*., 2024).

### Vector construction

See Table S1 for all primer sequences. To construct *pMYB305a::GFP*, a 2.14‐kb promoter region upstream of *MYB305a* was cloned from the European aspen SwAsp036 genotype using the primers proPtMYB305a_F_attB1/R_attB2 and ligated into pFASTG04 to drive GFP‐GUS expression. To construct *p35S::MYB305a*, the 1.204‐kb coding sequence of *MYB305a* without the stop codon was cloned from the European aspen SwAsp030 genotype using the primers PtMYB305a_CDS_F_attB1/R_attB2 and ligated into pFASTR05 to fuse with GFP, driven by the 35S promoter. A clustered regularly interspaced short palindromic repeats (CRISPR)/Cas9 approach (based on Xing *et al*., 2014) was used to generate the *myb305a* single and *myb305ab* double mutants. sgRNA cassettes targeting *MYB305a* and/or *MYB305a*/*b* were amplified from pCBC‐DT1T2 and/or pCBC‐DT1T2/DT2T3/DT3T4 using the primer pairs PtMYB305a_sgRNA1_T1F/sgRNA2_T2R and/or PtMYB305a_sgRNA1_T1F/DT1‐R, PtMYB305a_sgRNA2_T2F/DT2‐R, and PtMYB305b_sgRNA1_T3F/sgRNA3_T4R, and ligated into pHSE401 using the Golden Gate reaction based on the following procedures: 2 μl of pHSE401, 2–6 μl of sgRNA cassette fragment, 1.5 μl of 10x FastDigest buffer (Thermo Fisher Scientific, Waltham, MA, USA), 1.5 μl of 10 mM ATP, 1 μl of 30 U μl^−1^ T4 DNA ligase (Thermo Fisher Scientific), and 1 μl of FastDigest Eco31I (Thermo Fisher Scientific), incubated at 37°C for 5 h, then 50°C for 5 min and finally 80°C for 10 min. The vectors were cloned into chemically competent TOP10 *Escherichia coli* and purified from transformants selected on 50 μg ml^−1^ spectinomycin (for pFAST vectors) or kanamycin (for pHSE401) using the QIAprep Spin Miniprep Kit (Qiagen), according to manufacturer's instructions. The amplified plasmids were then sent to Eurofins Genomics (Ebersberg, Germany) for sequencing, followed by transformation of *Agrobacterium tumefaciens*. *Populus* transformations were performed by the UPSC Poplar Transgenics Facility (Umeå, Sweden). For details on transformation procedures, see Methods S1. Functional analyses were performed in hybrid aspen clone T89 due to the recalcitrance of European aspen to transformation; although the orthologs show strong sequence conservation and comparable expression patterns, we cannot exclude quantitative background‐dependent effects.

### Selection of transformed lines

*pMYB305a::GFP* lines were screened by confocal laser scanning microscopy on the adaxial epidermis of the terminal leaves and the line with the strongest GFP fluorescence was used for further experiments. CRISPR/Cas9‐generated mutant lines of *MYB305a* (Potrx042905g00010) and *MYB305b* (Potrx058201g00030) in T89 were genotyped with Phire Plant Direct PCR Master Mix (Thermo Fisher Scientific) according to the manufacturer's instructions, using the primers PttMYB305a_2sgR_gF/2sgR_gR and PttMYB305b_4sgR_gF/4sgR_gR (Table S1). The PCR product sizes suggested that the expected large‐fragment deletions were generated for *MYB305a* but not for *MYB305b*. Products were extracted from the PCR gels using the QIAquick Gel Extraction Kit (Qiagen), cloned into chemically competent TOP10 *E. coli* with the Zero Blunt TOPO PCR Cloning Kit (Invitrogen), and purified from *E. coli* transformants selected on 50 μg ml^−1^ kanamycin using the QIAprep Spin Miniprep Kit (Qiagen), according to the instructions of the kit manufacturers. The amplified plasmids were then sent to Eurofins Genomics for sequencing to determine actual DNA sequence alterations. The CLC Main Workbench (Qiagen) software was used for sequence alignments. For further experiments, lines of *myb305a* were selected in which both alleles of *MYB305a* contained a large‐fragment deletion at the expected site. Lines of *myb305ab* were selected in which both alleles of *MYB305a* and *MYB305b* contained either a large‐fragment deletion, a large‐fragment inversion, or indels that were expected to result in a frameshift mutation, at the expected sites. *p35S::MYB305a* lines were screened by reverse transcription quantitative polymerase chain reaction (RT‐qPCR) analysis of *MYB305a* expression levels in leaf samples from *in vitro* plants. Lines in which gene expression was enhanced compared with T89 were then grown in soil and analyzed again by RT‐qPCR for *MYB305a* expression levels in terminal leaves. Those lines in which gene expression was enhanced compared with T89 (2‐ to 10 000‐fold), without affecting plant height, were selected for further experiments.

### RT‐qPCR

RNA was extracted using the RNeasy Plant Mini Kit (Qiagen) according to the manufacturer's instructions, with minor adjustments. Leaf blade samples, excluding the petiole and mid‐vein, were ground in liquid nitrogen using a pestle and mortar and no more than 100 mg frozen ground tissue was used per sample for RNA extraction. The spin column membrane washing step was split into two washes of half the suggested buffer volume, with a 15‐min treatment with 80 μl RQ1 DNase (Promega) in between the washes. RNA concentrations were measured with a NanoDrop (Thermo Fisher Scientific), and cDNA was prepared from 1 μg RNA using the iScript cDNA Synthesis Kit (Bio‐Rad) according to the manufacturer's instructions. PCR was performed with cDNA using DreamTaq reagents (Thermo Fisher Scientific) and the primers PttMYB305a_RT‐qPCR_F1/R1 for visualization of amplified products in an agarose gel. RT‐qPCR was performed on a CFX96 Touch real‐time PCR detection system (Bio‐Rad) using Sso Advanced SYBR Green Supermix (Bio‐Rad) and the primers PttMYB305a_RT‐qPCR_F2/R2 for *MYB305a* (Potra2n8c18226) in European aspen, PttMYB305a_RT‐qPCR_F1/R1 for *MYB305a* (Potrx042905g00010) in T89 and PtUBQ_qRT‐PCR_F/R for the *UBIQUITIN* house‐keeping gene (designed for Potra2n1c3634 in European aspen). See Table S1 for primer sequences. The $2^{-\Delta \Delta C_{t}}$ method (Livak & Schmittgen, 2001) was used to calculate relative gene expression.

### Phylogenetic tree construction

The phylogenetic relationships of *P. tremula* MYB305a with its orthologs in Arabidopsis (*Arabidopsis thaliana* (L.) Heynh.) are taken from the pregenerated phylogenetic trees constructed as part of a large *Picea abies* (Norway spruce) and *Pinus sylvestris* (Scots pine) genome project (Ahlgren Kalman *et al*., 2025). For the phylogenetic relationships of *P. tremula* MYB305a with its orthologs in the T89 hybrid (*P. tremula* × *tremuloides*), all 281 annotated MYB transcription factor protein sequences (148 Potra and 133 Potrx loci) were retrieved from the respective genome annotations. Pairwise distances between sequences were calculated from tetrapeptide (4‐mer) amino acid composition vectors using cosine dissimilarity. The 35 sequences most similar to Potra2n8c18226 were selected for visualization. This subset was clustered using the Unweighted Pair Group Method with Arithmetic Mean (UPGMA), and the resulting tree is shown. All analyses were performed in Python using NumPy and SciPy.

### *k*‐means clustering and variance partitioning

The SwAsp individuals were clustered based on all cell shape and size features with *k‐*means cluster analysis using the kmeans function in R with nstart = 25. The data were first tested with the silhouette, gap statistic (clusGap), and elbow methods in the cluster package in R (Maechler *et al*., 2025) to determine the optimal number of clusters. We used genotype median values of published foliar carbon isotope discrimination data (Soolanayakanahally *et al*., 2015) as a proxy water‐use efficiency indicator for the SwAsp genotypes. The bioclimatic variables ‘mean annual precipitation’ and ‘precipitation of the driest month’ averaged for the years 1970–2000 were extracted from WorldClim (Hijmans, 2017) for the original SwAsp genotype sampling sites at 2.5 min resolution. Cluster membership assigned to SwAsp genotypes from the *k‐*means cluster analysis was used as a factor to partition the variance for carbon isotope discrimination, as well as annual precipitation, with separate tests for each variable. A Spearman rank correlation matrix was used to investigate the relationship between average precipitation metrics, latitude, and longitude for the original SwAsp sampling sites.

### Statistical analyses

Additional statistical analyses to those described above were performed using the software JMP and R and the sample sizes and statistical tests used are described in each relevant figure legend.

## Results

### Variation in complexity of leaf pavement cell shape among European aspen genotypes in the field

To investigate the variability of leaf pavement cell shape complexity within one tree species, we took advantage of the SwAsp collection of over 100 genotypes of European aspen that were collected from throughout Sweden and planted in a common garden (Luquez *et al*., 2008). We harvested leaves from the adult trees (so‐called preformed leaves, since they emerge from buds each Spring) during the summer, imaged the adaxial leaf blade surface, and analyzed several shape‐ and size‐related features of the pavement cells. The shape‐related traits included circularity (measure of deviation from a perfect circle), branch count (number of branches in the cell skeleton), lobe number (number of cell outgrowths), solidity (ratio of cell area to area of the cell's convex hull, a convex polygon fitting around the cell), and lobeyness (ratio of cell perimeter to convex hull perimeter; Möller *et al*., 2017; Sapala *et al*., 2018). The size‐related traits included nonlobe area (cell area excluding lobes), cell area, perimeter, and length and width (lengths of the major and minor axes, respectively, of an ellipse fitting around the cell; Möller *et al*., 2017). Nonlobe area and perimeter might also be considered as being related to cell shape. We observed that all of these shape‐ and size‐related traits varied among the SwAsp genotypes (Dataset S1). For example, mean solidity varied from a minimum of 0.855 to a maximum of 0.962, while mean perimeter varied from a minimum of 122.4 μm to a maximum of 194.5 μm. We then focused our attention on circularity, branch count, lobe number, and area, and found clear variation in these features among the genotypes (Figs 1, S1a–c). Mean circularity ranged from 0.57 to 0.74, while mean branch count ranged from 2.2 to 4.3, and mean lobe number ranged from 4.6 to 8.1. Finally, mean area ranged from 804 to 1846 μm^2^. We next selected four representative genotypes each for more complex (low circularity and high branch count and lobe number) and less complex (high circularity and low branch count and lobe number) pavement cell shape for later studies. These genotypes are indicated in magenta and cyan, respectively, in Figs 1(a) and S1(a–c) and with the same color coding in subsequent figures and are shown in more detail in Figs S2(a–d) and S3(a–h). We also noted that pavement cell shape was generally not highly complex, even in the genotypes with more complex cell shape (Fig. S3a–h). Many genotypes with simple cell shapes had small cells, and vice versa (Figs 1, S1a–c). However, this was not always the case. For example, the genotypes SwAsp057 and 073 displayed larger cells with simpler cell shapes, while SwAsp078 and 088 displayed smaller cells with more complex cell shapes (Figs 1, S1a–c).

**Fig. 1:**
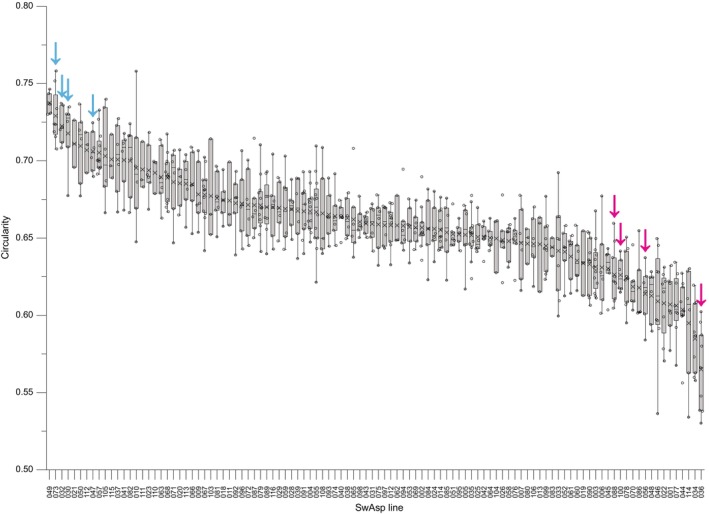
Pavement cell shape complexity varies among genotypes of Swedish Aspen (SwAsp) in the field. Circularity of pavement cells in the adaxial epidermis of preformed leaves in adult trees from the SwAsp collection of European aspen (*Populus tremula*) genotypes grown in the field. To ease visualization, each data point in the box plot represents the mean value of 70–160 cells measured per leaf rather than showing each measured cell; two to three leaves per tree and one to five trees per genotype were analyzed. Those genotypes selected as representatives of more and less complex cell shapes for further studies with terminal leaves of juvenile trees are indicated with magenta and cyan arrows, respectively. In the box plot, whiskers extend to the maximum/minimum values excluding outliers, a horizontal line indicates the median and x indicates the mean.

Since there is a reference genome assembly for European aspen (Lin *et al*., 2018; Robinson *et al*., 2024) and the genomes of most of the SwAsp genotypes have been fully resequenced (Wang *et al*., 2018), we took a GWAS approach with the aim of uncovering SNPs significantly associated with the cell shape traits. We identified a T‐to‐C SNP (chr8_11748682_C_T) in the sequence of a MYB transcription factor gene, *MYB305a* (Potra2n8c18226), that was significantly associated with increased branch count with additive effects (Figs 2a, S4a). This SNP explained 30% of the variation in branch count across the genotypes included in the GWAS and besides being the highest‐ranked SNP associated with branch count in terms of *P*‐value, it ranked number 233 for circularity, 685 for lobe number, 23 for solidity and 11 for lobeyness (Dataset S2). Among the size‐related features, the candidate SNP ranked number 120 for perimeter and 2805 for length and was not ranked among the SNPs in the top 1000 genes for nonlobe area, cell area or width (Dataset S2). The majority of the SwAsp genotypes were homozygous for the reference T allele for the candidate SNP, and they were generally classified in a less complex category of pavement cell shape, with lower branch count, lobe number and lobeyness, and with higher circularity and solidity than the other genotypes (Figs 2b, S3b–e). Another large group of genotypes were heterozygous for the T‐to‐C allele, and they could generally be seen to display higher branch count, lobe number and lobeyness, as well as lower circularity and solidity than the previous category (Figs 2b, S4b–e). Finally, two genotypes were homozygous for the alternative C SNP allele, and these were among the most complex cell shape genotypes, with among the highest branch count, lobe number and lobeyness values, and among the lowest circularity and solidity values of all the genotypes (Figs 2b, S4b–e). For the size‐related features, including nonlobe area, cell area, perimeter, length and width, the genotypes tended to overlap more than for the shape‐related features, although the two C/C genotypes did have somewhat larger cells than many of the other genotypes (Fig. S4f–j). These results suggest that *MYB305a* may be involved in regulating pavement cell shape in European aspen leaves.

**Fig. 2:**
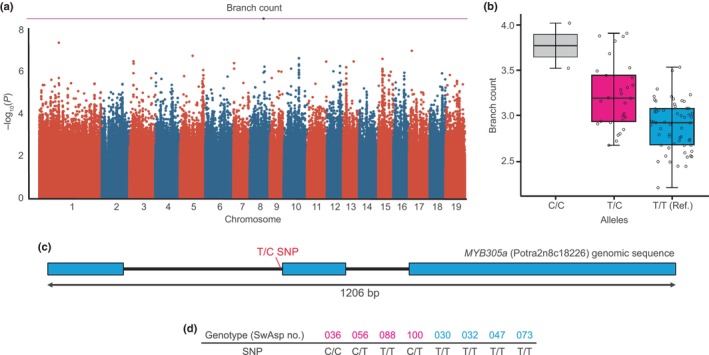
Identification of a single‐nucleotide polymorphism (SNP) in *MYB305a* associated with pavement cell shape complexity in European aspen. (a) Manhattan plot of the *P*‐values obtained for branch count in the genome‐wide association study (GWAS), in which a T‐to‐C SNP in the transcription factor *MYB305a* (Potra2n8c18226) was identified as being significantly associated with pavement cell branch count among the Swedish Aspen (SwAsp) collection of European aspen (*Populus tremula*) genotypes grown in the field. (b) Branch count of pavement cells in the SwAsp genotypes analyzed, grouped according to the alleles of the candidate SNP present in the genotypes. In the box plot, whiskers extend to the maximum/minimum values excluding outliers and a horizontal line indicates the median. (c) Position of the candidate SNP in the first intron of the *MYB305a* gene in European aspen. Introns are shown as black lines and exons as cyan boxes. (d) Alleles of the candidate SNP in the SwAsp genotypes selected as representatives of more (in magenta) and less (in cyan) complex cell shape.

The broad‐sense heritability of all the pavement cell traits was high, especially so for most of the shape‐related features (Fig. S5a), indicating high power for the GWAS to detect associations even for small‐effect alleles, and suggesting that environmental effects on the traits were relatively small. Genetic correlations of the cell features revealed the presence of two distinct axes of variation, visualized by two blocks in the heat map of genetic correlations – one representing shape simplicity traits (solidity and circularity) and the other representing shape complexity and size traits (all other shape‐related features and all size‐related features; Fig. S5b). While solidity and circularity were strongly positively correlated with each other, they were somewhat negatively correlated with the size‐related traits. Intuitively, circularity and solidity were strongly negatively correlated with the shape‐related traits lobe number, branch count, and lobeyness, which in turn were somewhat positively correlated with the size‐related traits (Fig. S5b). Overall, there is a genetic correlation between pavement cell size and shape, with more complex cells tending to be larger.

The identified candidate SNP [chr8_11748682_C_T] is located at the end of the first intron of *MYB305a* (Fig. 2c) and the T‐to‐C variation is present in three of the four previously selected representative genotypes for more complex pavement cell shape, while being absent from all four representatives for less complex shape (Fig. 2d). To investigate whether the SNP may interfere with RNA splicing, we measured expression of *MYB305a* in the representative SwAsp genotypes for more and less shape complexity with two primer pairs, one of which spanned the first intron and one of which did not span an intron (Fig. S6a) and compared the results. For the primer pair that spanned the first intron, a product of the expected size of *c*. 200 bp was amplified from cDNA in all lines, although additional, larger products were also amplified in several of the lines, possibly representing alternative transcripts (Fig. S6b). The relative gene expression levels measured by RT‐qPCR using the other primer pair (Fig. S6c) generally matched the abundance of the product detected using the primer pair spanning the first intron (Fig. S6b), suggesting that the candidate SNP does not interfere with RNA splicing. While we infer that the candidate SNP or a linked variant may influence *MYB305a* regulation, the GWAS association remains correlative. Rather than attempting to functionally characterize the SNP, which would be extremely challenging in this species, we took a GWAS‐to‐gene approach, choosing to focus our efforts on characterizing a biological role for *MYB305a* in aspen pavement cell shape acquisition. The closest orthologs of European aspen *MYB305a* (Potra2n8c18226) and its closest homolog *MYB305b* (Potra2n10c21884) in Arabidopsis are *MYB305* (also known as *MYB71*; AT3G24310) and *MYB79* (AT4G13480; Fig. S6d), which are nuclear‐localized transcriptional activators that show enhanced expression in response to abscisic acid (ABA) treatment (Cheng *et al*., 2022). Furthermore, *MYB305*/*MYB71* has been shown to be involved in ABA and abiotic stress responses (Cheng *et al*., 2022).

### Increased complexity of pavement cell shape from field to glasshouse conditions

To investigate the robustness of pavement cell shape complexity in the SwAsp lines grown in different conditions, leaves from juvenile trees (so‐called terminal leaves formed at the terminal meristem and emerging directly from the stem) grown in controlled glasshouse conditions were used. We also took the opportunity to analyze pavement cell shape complexity and area in terminal leaves of different developmental stages for the eight representative SwAsp genotypes of more and less complex pavement cell shape. Adaxial pavement cell shape complexity increased, with circularity decreasing and both branch count and lobe number increasing over the terminal leaf series as the leaves developed and the cells grew in area (Fig. 3a–d). By Leaf 17, there was a clear separation of the genotypes into two groups of more or less complex cell shape for all the shape features measured (Figs 3a–c, S7a–h).

**Fig. 3:**
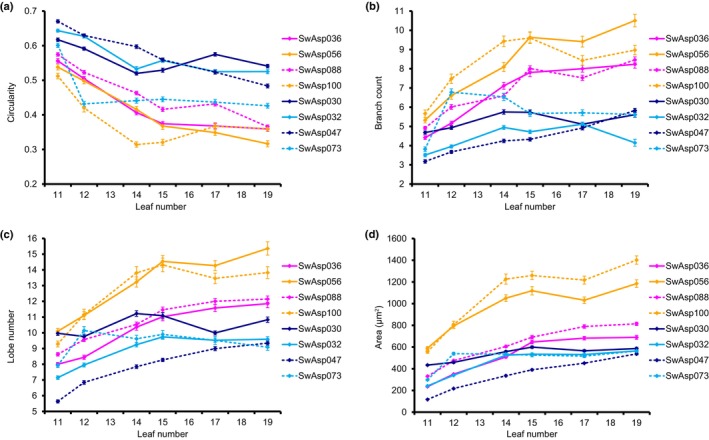
Pavement cell shape complexity in terminal leaves of juvenile European aspen in the glasshouse. (a–d) Circularity (a), branch count (b), lobe number (c), and area (d) of pavement cells in the adaxial epidermis of terminal leaf numbers 11–19 in juvenile trees from the Swedish Aspen (SwAsp) collection of European aspen (*Populus tremula*) genotypes grown in the glasshouse. Leaf number was counted from the top down, with number 1 being the youngest leaf. Each data point represents the mean value of 30–250 cells in total, measured across one leaf per tree and three trees per genotype. Error bars represent SE of the mean. SwAsp genotypes from the more and less complex preformed leaf pavement cell shape groups are shown in magenta/orange and cyan/dark blue colors, respectively.

Importantly, the lines followed the same trend in shape complexity for terminal leaves as was found for preformed leaves, with those genotypes that were the most or least complex for terminal leaf pavement cell shape also being the most or least complex for preformed leaves, respectively (Fig. 3a–c compared with Fig. S2a–c), indicating high robustness of these traits. However, in the more developed terminal leaves, the pavement cells were generally more complex in shape, with lower circularity and higher branch count and lobe number than in the preformed leaves of the same SwAsp genotypes in the field (Figs 3a–c, S7a–h compared with Figs S2a–c, S3a–h). The SwAsp lines with more complex pavement cell shape also displayed larger pavement cells in terminal leaves (Fig. 3d). However, despite being more complex in shape, pavement cell area was smaller in the mature terminal leaves from juvenile trees in the glasshouse than in the preformed leaves from adult trees in the field for all genotypes except SwAsp100 (Fig. 3d compared with Fig. S2d).

### Genetic modifications of *MYB305a* suggest its involvement in pavement cell shape regulation

To validate the GWAS results and confirm a role for *MYB305a* in pavement cell shape regulation in European aspen leaves, we intended to generate *MYB305a* native promoter‐driven GFP reporter lines, CRISPR/Cas9‐generated *MYB305a* large‐fragment deletion mutants and *MYB305a* over‐expressor lines, using SwAsp036 and 030 as the genetic backgrounds, representing genotypes with the most and least complex cell shapes, respectively. However, since no plants from these genotypes survived the procedures, we instead turned to clone T89 of hybrid aspen (*Populus tremula* × *tremuloides*), which is known to be easily transformed. A phylogenetic analysis of the full MYB transcription factor gene family in European and hybrid aspen, encompassing 281 annotated family members (148 *P. tremula* [Potra] loci and 133 *P. tremula* × *tremuloides* [Potrx] loci), revealed that the closest T89 orthologs of *P. tremula MYB305a* (Potra2n8c18226) are Potrx042905g00010 and Potrx058201g00030, with Potrx058201g00030 being closer to *P. tremula MYB305b* (Potra2n10c21884; Fig. S8a). These genes form a well‐supported clade, demonstrating that functional work carried out in T89 should directly reflect the biology of the *P. tremula* gene, although we acknowledge that species‐ or background‐specific effects cannot be completely excluded. T89 is homozygous for the reference T SNP allele of *MYB305a* (Potrx042905g00010) at the position of interest. We confirmed that developing terminal leaves from juvenile T89 trees in the glasshouse display similar progression of cell shape complexity acquisition and cell growth during leaf development as the SwAsp genotypes (Fig. S8b–e compared with Fig. 3a–d) and then proceeded with genetic modifications in T89.

We obtained six *pMYB305a::GFP* lines in T89 in which we investigated GFP fluorescence. In three of the lines, we were not able to visualize any GFP fluorescence in the terminal leaves, while in two of the lines (Lines 8 and 11), we observed very faint GFP fluorescence in the adaxial pavement cells (Fig. S9a). In one line (Line 4), we found stronger, more clearly visible GFP fluorescence in the pavement cells (Fig. S9a). These observations suggested that while the *MYB305a* promoter is indeed expressed in aspen pavement cells, it is likely expressed at a rather low level. We next investigated the GFP expression patterns in a series of terminal leaves of different developmental stages in juvenile trees of *pMYB305a::GFP* Line 4 grown in the glasshouse. While the expression level was very low in younger leaves, numbers 9–13, it was not consistent throughout the epidermis, with clearly enhanced expression being observed in specific pavement cells across the tissue (Fig. 4a–c). A striking increase in expression was observed across the epidermis in the older leaves, numbers 15–19 (Fig. 4d–f), compared with the younger leaves.

**Fig. 4:**
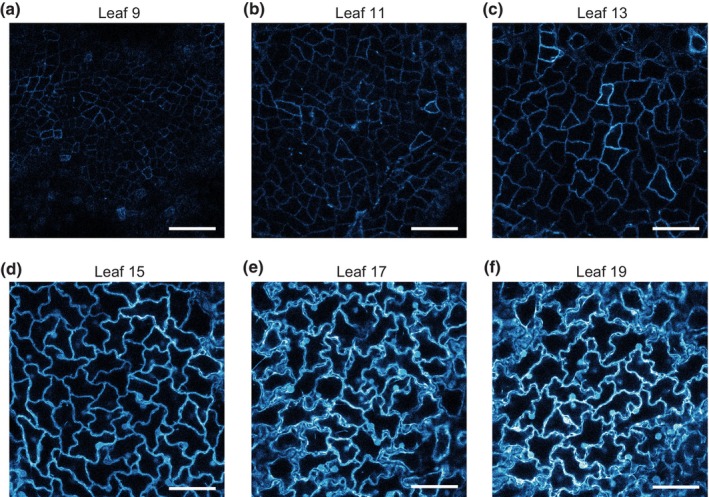
*MYB305a* promoter expression increases in pavement cells as they grow and acquire their complex shape. (a–f) Representative confocal microscopy images of GFP fluorescence in pavement cells in the adaxial epidermis of terminal leaf numbers 9–19 in *pMYB305a::GFP* Line 4 of hybrid aspen (*Populus tremula* × *tremuloides*; T89) grown in the glasshouse. Increasing GFP fluorescence is represented by a cyan‐to‐white color scale. Bars, 50 μm.

Using CRISPR/Cas9 technology, we generated *MYB305a* large‐fragment deletion lines in T89 and identified six lines in which deletions of 371–396 bp in both alleles in the region of the candidate SNP location were confirmed by sequencing (Fig. S9b,c). We also generated five over‐expressor lines of *MYB305a* in T89 and confirmed highly variable *MYB305a* over‐expression levels in these lines by RT‐qPCR in terminal leaves (Fig. S9d). We also noticed that *MYB305a* expression was rather low in the leaves of T89 controls, only becoming detectable after 30 amplification cycles (compared with 19 cycles for the house‐keeping gene). For the four deletion lines that we grew in the glasshouse, *myb305a* Lines 4, 20, 24, and 29, we did not observe any significant difference in shoot height compared with T89 after 8 wk of growth in soil (Fig. S10a,b). Of the over‐expressor lines, *p35S::MYB305a* Line 1, which had the highest over‐expression level of *c*. 54 000‐fold (Fig. S9d), was severely stunted in shoot growth after 8 wk of growth in soil, with much smaller leaves and significantly shorter shoots than T89 (Fig. S10c,d). *p35S::MYB305a* Line 5, which displayed the next‐highest over‐expression level of *c*. 11 000‐fold (Fig. S9d), had somewhat smaller leaves than T89 (Fig. S10c,d).

We then selected three of the deletion lines, *myb305a* Lines 4, 20, and 24, and three of the over‐expressor lines (excluding the line with highest over‐expression to avoid pleiotropic effects), *p35S::MYB305a* Lines 3, 4, and 5, for studies of adaxial pavement cell shape in terminal leaves of juvenile plants grown in the glasshouse. Compared with T89, *myb305a* Lines 4 and 20 displayed slightly but significantly increased circularity, decreased branch count and decreased lobe number in the pavement cells, while Line 29 was unaffected in these features compared with T89 (Fig. 5a–c). The three *p35S::MYB305a* lines all showed significant increases in circularity and decreases in branch count and lobe number compared with T89 and these effects were generally somewhat stronger than those observed in the *myb305a* lines (Fig. 5a–c). For circularity and branch count, the difference in the *p35S::MYB305a* lines to the control was related to the level of over‐expression (Fig. S9d), with highest over‐expression leading to strongest difference in circularity/branch count compared with the control (Fig. 5a–c). Finally, cell area was only significantly affected in one of the *myb305a* lines, in which it was slightly decreased (Fig. 5d). Together, these results strongly suggest that *MYB305a* is involved in pavement cell shape regulation in aspen leaves independently of any potential role in cell expansion. Interestingly, *myb305a* Lines 20 and 29 had deletion lengths expected to result in frameshift mutations, while Line 4 did not, which suggests that the deleted region itself, which spanned the candidate SNP location, is important for this function of MYB305a. Since both *myb305a* and *p35S::MYB305a* lines generally displayed decreased pavement cell shape complexity without cell area being affected, we assume that exact, fine‐tuned levels of MYB305a, likely with specific, precise timing of expression during development, must be important for promoting cell shape acquisition independently from general cell growth regulation. An alternative possibility is that MYB305a may form a protein complex whose function is disrupted by abnormal MYB305a levels.

**Fig. 5:**
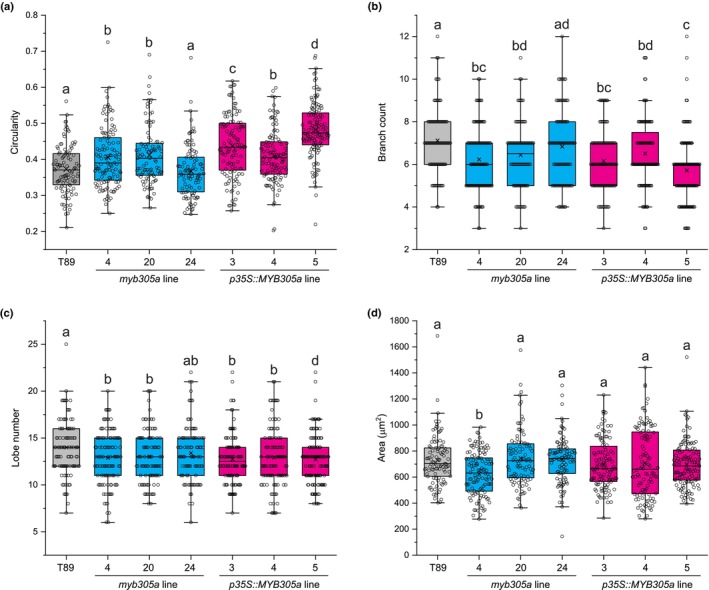
Pavement cell shape features of *MYB305a* CRISPR mutant and over‐expressor lines. (a–d) Circularity (a), branch count (b), lobe number (c), and area (d) of pavement cells in the adaxial epidermis of terminal leaf number 17 in *MYB305a* CRISPR mutant (*myb305a*, in cyan) and over‐expressor (*p35S::MYB305a*, in magenta) lines of hybrid aspen (*Populus tremula* × *tremuloides*; T89) grown in the glasshouse. Each data point in the box plot represents one of 95–125 cells in total measured across one leaf per tree and three trees per genotype. Different letters indicate significantly different distributions at *P* < 0.05 (Wilcoxon rank sum test). In the box plots, whiskers extend to the maximum/minimum values excluding outliers; a horizontal line indicates the median, and x indicates the mean.

There is a close homolog of *MYB305a* in European aspen, Potra2n10c21884, which we called *MYB305b* (Fig. S6d) and which corresponds to Potrx058201g00030 in hybrid aspen (Fig. S8a). We therefore also attempted to generate double *MYB305a MYB305b* CRISPR/Cas9 large‐fragment deletion lines in T89. Using the same strategy as before, with the same guide RNAs, we were successful in generating large‐fragment deletions or inversions in *MYB305a* in most of the lines we generated, as confirmed by sequencing (Fig. S11a). However, our attempts to generate large‐fragment deletions in *MYB305b* (Fig. S11b) generally only resulted in one‐nucleotide indels around the sites of expected DNA breaks in most of the lines (Fig. S11c). For the five lines that we grew in the glasshouse, *myb305ab* Lines 25, 26, 29, 32, and 36, we observed an apparent slight decrease in shoot height after 8 wk of growth in soil, but there were no statistically significant differences to T89 shoot height (Fig. S11d,e). We randomly selected three of the lines, *myb305ab* Lines 26, 32, and 36, in which we confirmed a large‐fragment deletion or inversion in both alleles of *MYB305a* and expected a frameshift mutation in both alleles of *MYB305b*, for studies of adaxial pavement cell shape in terminal leaves of juvenile plants grown in the glasshouse. Compared with T89, two of the *myb305ab* lines displayed slightly but significantly increased circularity in the pavement cells, with Line 36 being the most affected (Fig. S12a). The same two lines displayed significantly decreased branch count and Line 36 additionally displayed significantly decreased lobe number and cell area (Fig. S12b–d). These results were similar to those found for the *myb305a* lines and the lack of an additive effect in *myb305ab* compared with *myb305a* suggests that *MYB305b* might not play a functionally redundant role with *MYB305a* in the regulation of pavement cell shape in aspen leaves.

### *MYB305a* is drought stress‐responsive and may regulate pavement cell shape response to abiotic stress

The closest homolog of aspen *MYB305a* in Arabidopsis, *MYB305* (also known as *MYB71*; AT3G24310; Fig. S6d), has been shown to be upregulated in response to ABA treatment and to positively regulate a number of stress response‐related genes (Cheng *et al*., 2022). Since ABA is known to mediate drought stress responses in plants (Aslam *et al*., 2022) and the expression of many other MYB transcription factors is regulated by drought (Baldoni *et al*., 2015), we investigated whether drought stress would affect *MYB305a* expression in T89 terminal leaves. After a moderate drought stress treatment of several days on *pMYB305a::GFP* Line 4, which resulted in a significant reduction in shoot height and some leaf wilting in the lower half of the shoot (Fig. S13a,b), we imaged the epidermis in terminal leaves of different ages. In leaf numbers 9–13, in which the cells developed from rather small and square‐shaped to much larger with more complex shape, a clear enhancement of GFP fluorescence was visible in the drought‐stressed trees compared with the control trees (Fig. 6a). Simultaneously, the drought stress also induced a significant increase in cell shape complexity compared with the controls in these younger leaves (Fig. 6b–d). Surprisingly, once GFP fluorescence had reached around the same levels in the controls as in the drought treatments, from around Leaf 15 and upwards (Fig. 6a), the drought stress began to have the opposite effect on cell shape, resulting in a significant reduction in cell shape complexity compared with the controls (Fig. 6b–d). These results imply that *MYB305a* promoter expression is strongly induced by drought stress in younger aspen leaves and that *MYB305a* may be involved in enhancing the development of pavement cell shape in these young leaves in response to the drought stress. However, in older leaves, in which *MYB305a* promoter expression is already much higher than younger leaves in the controls, the later stages of pavement cell shape development are repressed by the drought stress. These results agree with the idea that fine‐tuned MYB305a levels with proper timing may be important for regulating the correct pavement cell shape complexity level according to the stage of development and environmental conditions.

**Fig. 6:**
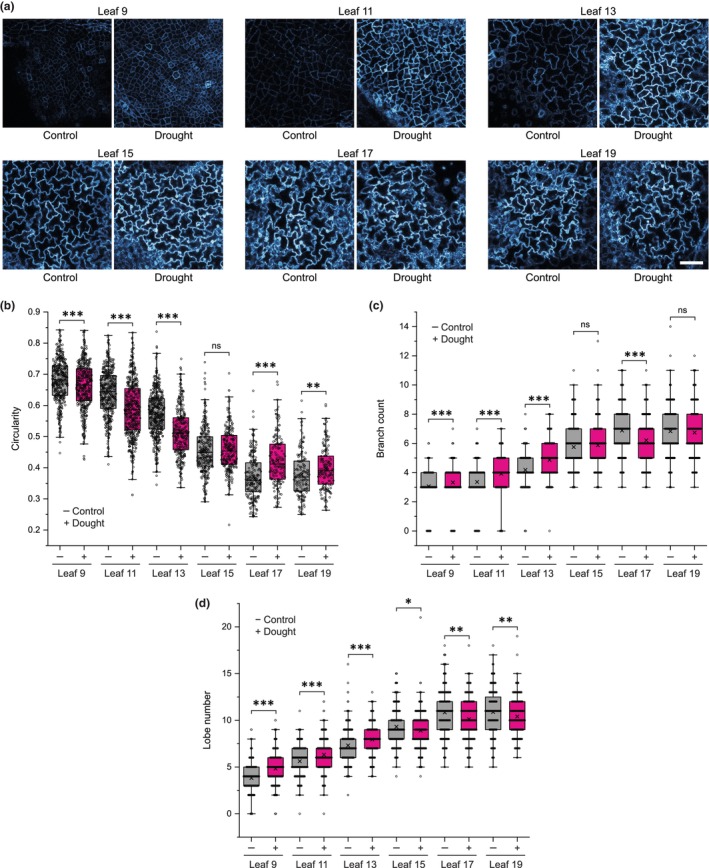
*MYB305a* promoter expression and pavement cell shape respond to drought stress. (a) Representative confocal images of GFP fluorescence in pavement cells in the adaxial epidermis of terminal leaf numbers 9–19 in *pMYB305a::GFP* Line 4 of hybrid aspen (*Populus tremula* × *tremuloides*; T89) grown in control or drought conditions in the glasshouse. Increasing GFP fluorescence is represented by a cyan‐to‐white color scale. Identical acquisition settings were used for each image. Bar, 50 μm. (b–d) Circularity (b), branch count (c), and lobe number (d) of pavement cells in the adaxial epidermis of terminal leaf numbers 9–19 in *pMYB305a::GFP* Line 4 of hybrid aspen (T89) grown in control or drought conditions in the glasshouse (see (a) for representative images). Each data point in the box plot represents one of 156–485 cells in total measured across one leaf per developmental stage per tree and three trees per treatment. Asterisks indicate significantly different distributions between the control and drought treatment according to the Wilcoxon rank sum test (ns, not significant; *, *P* < 0.05; **, *P* < 0.01; ***, *P* < 0.001). In the box plots, whiskers extend to the maximum/minimum values excluding outliers, a horizontal line indicates the median, and x indicates the mean.

We next tested the effects of the drought treatment on cell shape features in two each of our *myb305a* deletion and *p35S::MYB305a* over‐expressor lines. The drought treatment resulted in significantly reduced shoot height in all the lines as well as in T89 control trees (Fig. S14a,b). We compared any drought stress‐induced changes in cell shape features in Leaf 17 of the transformed lines to that of T89. In T89, pavement cell circularity was increased 1.2‐fold by the drought stress compared with control conditions (Fig. 7a; note that ratios of drought‐to‐control circularity are shown). Interestingly, both of the *myb305a* lines were resistant to this effect of drought stress on circularity, while the *p35S::MYB305a* lines were affected in circularity to the same degree as T89 (Fig. 7a). In T89, the drought stress resulted in *c*. 0.9‐fold decreases in pavement cell branch count and lobe number compared with control conditions (Fig. 7b,c; note that ratios of drought‐to‐control branch count and lobe number are shown). While one of the *myb305a* lines was affected to a similar extent as T89, the other *myb305a* line was resistant to this effect of drought stress on both branch count and lobe number (Fig. 7b,c). Conversely, while one of the *p35S::MYB305a* lines was affected to a similar extent as T89, the other *p35S::MYB305a* line was over‐sensitive to the effect of drought stress on branch count and lobe number, in that they were reduced more than in T89 (Fig. 7b,c). Although these results are not unequivocal, taken together with the effects of drought stress on inducing *MYB305a* promoter expression, they imply that *MYB305a* may play a role in the response of pavement cell shape complexity to drought stress in aspen leaves.

**Fig. 7:**
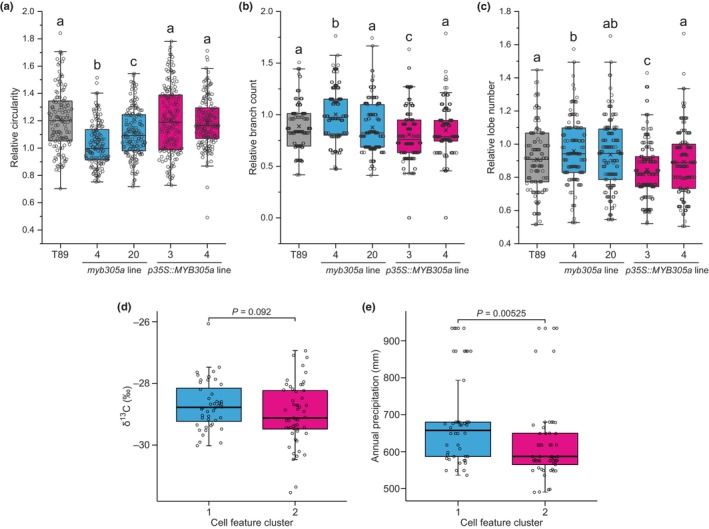
*MYB305a* may play a role in the response of pavement cell shape to drought stress and pavement cell shape is correlated with water use and availability. (a–c) Relative drought:control circularity (a), branch count (b), and lobe number (c) of pavement cells in adaxial epidermis of terminal leaf number 17 in *MYB305a* CRISPR mutant (*myb305a*, in cyan) and over‐expressor (*p35S::MYB305a*, in magenta) lines of hybrid aspen (*Populus tremula* × *tremuloides*; T89) grown in the glasshouse. Relative cell shape features were calculated as the drought:control ratio; each data point in the box plot represents the ratio of one of 125–160 cells in total measured across one leaf per tree and three trees per genotype in drought stress conditions, compared with the mean control (no drought stress) value. Different letters indicate significantly different distributions at *P* < 0.05 (Wilcoxon rank sum test). In the box plots, whiskers extend to the maximum/minimum values excluding outliers, a horizontal line indicates the median, and x indicates the mean. (d, e) Cluster assignment of the Swedish Aspen (SwAsp) genotypes of European aspen (*Populus tremula*) from the *k‐*means cluster analysis was used as a factor to partition the variance for foliar carbon isotope discrimination (delta^13^C) (d) and annual precipitation averaged over 30 yr (e) at the original sampling sites. The *P*‐values indicated were calculated using the Wilcoxon rank sum test. In the box plots, whiskers extend to the maximum/minimum values excluding outliers, and a horizontal line indicates the median.

We next turned our attention back to the SwAsp population, since we showed that the genotypes naturally vary in pavement cell shape complexity and earlier work has estimated the water‐use efficiency of the different genotypes (Soolanayakanahally *et al*., 2015). We performed *k*‐means clustering to partition the data into an optimal number of clusters and observed that our selected representative genotypes of more and less complex pavement cell shape were divided between the two resulting clusters (Fig. S15a). We then color‐coded the branch count values of the genotypes measured earlier according to their clusters (Fig. S15b). This indicated that the genotypes were generally separated according to pavement cell shape complexity by the clustering. We therefore statistically compared the water‐use efficiency of the genotypes (estimated by a measure of the foliar carbon isotope ratio or delta^13^C) between the two clusters (Fig. 7d). Although the difference between the clusters was not statistically significant, the rather low *P*‐value suggests the possibility of a mild negative correlation between pavement cell shape complexity and water‐use efficiency (Fig. 7d). Records of environmental variables at the original SwAsp sampling sites are available, including annual precipitation and precipitation of the driest month of the year, both of which we averaged over a 30‐yr period ending just 3 yr before the genotypes were originally sampled. We compared the average annual precipitation between the clusters and this time we found a highly significant difference, with the more complex cell shape cluster genotypes tending to originate from sites with less precipitation (Fig. 7e). Since the bioclimatic precipitation variables are presumably correlated, and also likely to be correlated with geographic location variables, we then generated a correlation heat map of annual precipitation, precipitation of the driest month of the year, latitude, and longitude of the original sampling sites (Fig. S16). The heat map confirmed a positive correlation between annual precipitation and precipitation of the driest month as well as a negative correlation between the precipitation variables and both latitude and longitude. Taken together, these results strengthen the idea that climatic variables affect the shape complexity of leaf pavement cells in the genotypes. Overall, our work suggests that the level of pavement cell shape complexity in aspen leaves results from a combination of evolutionary adaptation to environmental conditions such as water availability within a genotype, and developmental responses to local stresses such as drought in the individual tree during leaf growth. Furthermore, our results provide a foundation for future mechanistic studies on leaf pavement cell shape in aspen in response to environmental conditions, by implicating the involvement of the transcription factor MY305a.

## Discussion

Although the mechanisms for regulation of pavement cell shape complexity in aspen leaves are largely unknown, our work has provided an important foundation for understanding this process by revealing the involvement of the transcription factor MYB305a. Pavement cell shape is hugely variable both among and within plant species (Vőfély *et al*., 2019; Liu *et al*., 2021) and the biological reasons for the interdigitated, jigsaw puzzle‐like shape are therefore not entirely obvious. Enhanced integrity and reduced mechanical stress in the epidermis of a near‐isotropically expanding organ potentially exposed to a wide variety of environmental stresses are likely functions of this cell shape (Sapala *et al*., 2018; Bidhendi *et al*., 2023). The study of pavement cell shape regulation is therefore valuable because plasticity of the puzzle shape may be an important adaptation to environmental conditions that are likely to influence the mechanical stress patterns. Many plant species display widely different pavement cell morphologies in different growth conditions and/or different organs of the same plant (Vőfély *et al*., 2019; Liu *et al*., 2021; Trozzi *et al*., 2026). Interestingly, similarly to our findings for field‐grown and glasshouse‐grown European aspen, pavement cells in camphor tree (*Camphora officinarum*) were recently also shown to display much more complex, lobed shapes when the plants were grown indoors compared with when grown outdoors (Trozzi *et al*., 2026). In European aspen, fully developed terminal leaves are considerably larger in area than fully developed preformed leaves (Mähler *et al*., 2020) and larger leaves may be expected to result in more complex pavement cell shapes as a strategy to mitigate the mechanical stresses imposed by the more extensive near‐isotropic cell expansion in such organs (Trozzi *et al*., 2026).

Our work was facilitated by access to the SwAsp collection of European aspen genotypes, which allowed us to link a biological process to a natural population originating from native habitats, rather than an artificial environment. Over the last few years, the SwAsp collection has proven itself to be a highly valuable resource for tree‐related research in Sweden, convenient for investigating a diverse range of biological questions (Luquez *et al*., 2008; Robinson *et al*., 2012; Keefover‐Ring *et al*., 2014; Vain *et al*., 2019; Lihavainen *et al*., 2023; Luomaranta *et al*., 2024). We also detected correlations of water‐use efficiency and precipitation at the sampling sites of the SwAsp clones with pavement cell shape complexity. At first glance, the negative correlation of water‐use efficiency and precipitation with cell shape complexity may seem to contradict the effect of drought treatment on simplifying cell shape. However, the SwAsp experiments were performed with preformed leaves sampled from adult trees in the field, while the drought experiments were performed with terminal leaves from glasshouse‐grown juvenile trees, with the different environments and leaf types likely to affect the results. This apparent contradiction was also mirrored in the drought experiment on T89 leaves of different developmental stages, which surprisingly showed drought‐induced enhancement of shape complexity in younger leaves but simplification in older leaves. While these results suggest that pavement cell shape responds to water stress, there are likely to be many additional factors influencing the final cell shape.

The GWAS approach has proven a powerful strategy to reveal new molecular players in regulatory processes in *Populus* species. For example, using GWAS for measures of stem growth traits over time in a large population of Chinese white poplar (*P. tomentosa*) genotypes, a role for prolyl 4‐hydroxylase 9 was revealed and subsequently confirmed in the promotion of stem diameter growth (Xiao *et al*., 2023). A GWAS strategy was also successfully employed in natural populations of Chinese white poplar and Chinese cottonwood (*P. simonii*) to identify new molecular players in *Populus* leaf morphology (Liu *et al*., 2023). On the contrary, GWAS for leaf shape features in the SwAsp genotypes failed to reveal statistically significant SNP associations, which combined with further gene expression analyses suggested that leaf shape variation is a complex, omnigenic trait in European aspen (Mähler *et al*., 2020). In our study, we only found a single SNP that was significantly associated with pavement cell shape in the SwAsp genotypes, and only for one cell shape trait across all that we included, suggesting that leaf pavement cell shape in European aspen may be highly polygenic. While quantitative trait loci (QTLs) for epidermal cell area in leaves of black cottonwood (*P. trichocarpa*) and eastern cottonwood (*P. deltoides*) have been identified previously (Ferris *et al*., 2002), we are not aware of any work identifying QTLs or significant SNP associations by GWAS for shape features in *Populus* epidermal leaf cells. Importantly, despite only identifying one candidate SNP, we succeeded in its subsequent validation using transgenic approaches, supporting a role for *MYB305a* in aspen pavement cell shape regulation.

MYB305a is a member of the R2R3‐type MYB transcription factors, a large family including both activators and repressors of gene expression that are involved in many physiological processes (Stracke *et al*., 2001; Dubos *et al*., 2010). In black cottonwood, there are almost 200 R2R3‐type *MYB* genes, suggesting a diverse range of functions and implying a role for this family in the phenotypic diversity of *Populus* species (Wilkins *et al*., 2009). Transcription factors are themselves also subject to complex expressional regulation mechanisms and contain regulatory elements in their genes, which can occur in the introns, as exemplified by the floral identity transcription factor gene *AGAMOUS* in Arabidopsis (Kaufmann *et al*., 2010). The cell shape‐associated SNP we identified through our GWAS is located in the first intron of *MYB305a*, perhaps at a site particularly important for its temporal or spatial regulation, since overall expression levels appeared unaffected by the SNP. Since both deletion and over‐expression of the gene resulted in reduced pavement cell shape complexity, we cannot establish a direct mechanistic link between the specific intronic SNP and *MYB305a* regulation. Our study does not establish variant‐level causality; rather, it demonstrates that *MYB305a* itself plays a functional role in pavement cell shape complexity, consistent with its identification as a GWAS‐prioritized candidate gene. The cell shape regulatory pathways in which MYB305a is active are likely to be complex, in which precise levels of this transcription factor with exact timing are important for achieving the proper level of cell shape complexity needed according to the developmental context and environmental conditions.

To our knowledge, *MYB305a* has not previously been studied in *Populus* species, but investigations on its close homolog in Arabidopsis, *MYB305*/*MYB71*, revealed its function as a transcriptional activator in the nucleus, its upregulation in response to ABA treatment and its likely activity as a positive regulator of ABA response (Cheng *et al*., 2022). ABA is a well‐known mediator of stress‐responsive signaling (Aslam *et al*., 2022; Singh & Roychoudhury, 2023; Mo *et al*., 2024) and the authors found a small set of genes upregulated by *MYB505*/*MYB71*, of which the majority are known to be involved in stress responses (Cheng *et al*., 2022). Many R2R3‐type MYB family members are up‐ or downregulated by drought treatment and thought to play roles in drought stress responses in various different species (Villalobos *et al*., 2004; Cominelli *et al*., 2005, 2008; Seo *et al*., 2009; Yang *et al*., 2012; Zhang *et al*., 2012; Cao *et al*., 2013; Jin *et al*., 2025). The upregulated expression of several R2R3‐type MYB‐encoding genes in response to drought stress has recently been shown in black cottonwood (Zhang *et al*., 2023), and we demonstrated that *MYB305a* expression is also clearly upregulated in European aspen leaves upon drought stress. Overall, our results suggest that this transcription factor regulates drought stress‐induced changes in pavement cell shape complexity in aspen leaf epidermis.

A set of closely related R2R3‐type MYB family genes have already been reported to regulate epidermal cell shape in various organs across several species. Early work suggested that *MIXTA* in snapdragon (*Antirrhinum majus*) regulates the cone shape of epidermal cells in flower petals (Noda *et al*., 1994), which was later also shown for *MIXTA*‐related *MYB* genes in the same and other species, including Arabidopsis *MYB16* (Baumann *et al*., 2007). *MIXTA*, *MYB16*, and the close homolog *MYB106* in Arabidopsis have additionally been implicated in the regulation of leaf trichome shape (Glover *et al*., 1998; Jakoby *et al*., 2008; Gilding & Marks, 2010). Roles for *MYB16* and *MYB106* in cuticle biosynthesis have also been shown (Oshima *et al*., 2013), and more recently, *MYB16* was additionally suggested to regulate the division and differentiation of young stomatal lineage‐derived pavement cells in the cotyledon epidermis (Ho *et al*., 2021). These studies reveal that the *MIXTA*‐like R2R3‐type MYB transcription factors play multi‐functional roles in plant development but seem to be particularly important for regulation of epidermal cell shape. Our work specifies that *MYB305a* regulates pavement cell shape in European aspen leaves, but whether this role is specific to this cell type and species is still unknown. Many questions also arise regarding which molecular signaling pathways and mechanisms *MYB305a* acts in to regulate this process, and we look forward to the identification of interactors and targets of this transcription factor in aspen leaves in the future.

## Competing interests

None declared.

## Author contributions

SR designed the research. SL, SMD, KMR and ZR performed the research. SL, SMD, KMR, NRS and SR analyzed and interpreted the data. SMD wrote the manuscript with input from all authors. SL and SMD contributed equally to this work.

## Disclaimer

The New Phytologist Foundation remains neutral with regard to jurisdictional claims in maps and in any institutional affiliations.

## Supporting information


**Dataset S1** Pavement cell shape‐ and size‐related features of Swedish Aspen genotypes in the field.

**Dataset S2** Top‐ranked 1000 genes for each cell feature based on significance of the association of its SNPs with that feature.

**Fig. S1** Pavement cell shape complexity varies among genotypes of Swedish Aspen in the field.
**Fig. S2** Pavement cell features of representative SwAsp genotypes of more and less complex cell shape in the field.
**Fig. S3** Pavement cell images of representative SwAsp genotypes of more and less complex cell shape in the field.
**Fig. S4** Identification of a SNP in *MYB305a* associated with pavement cell shape complexity in European aspen.
**Fig. S5** Broad‐sense heritability and genetic correlations of the pavement cell features in European aspen.
**Fig. S6** Expression of *MYB305a* in SwAsp genotypes and the closest phylogenetic relatives of *MYB305a* in European aspen and Arabidopsis.
**Fig. S7** Pavement cell shape complexity varies among genotypes of juvenile European aspen in the glasshouse.
**Fig. S8** Closest phylogenetic relatives of *MYB305a* in European aspen and hybrid aspen clone T89 and pavement cell shape complexity in terminal leaves of juvenile T89 in the glasshouse.
**Fig. S9** Selection of genetically transformed *pMYB305a::GFP*, *MYB305a* CRISPR/Cas9 deletion and *p35S::MYB305a* over‐expressor lines in T89.
**Fig. S10** Shoot height in *MYB305a* CRISPR/Cas9 deletion and *p35S::MYB305a* over‐expressor lines in T89.
**Fig. S11** Line selection and shoot height in *MYB305a MYB305b* CRISPR/Cas9 deletion lines in T89.
**Fig. S12** Pavement cell shape features in *MYB305a MYB305b* CRISPR/Cas9 deletion lines in T89.
**Fig. S13** Effects of drought stress on shoot height and pavement cell shape features in T89 leaves of different developmental stages.
**Fig. S14** Effects of drought stress on shoot height in *MYB305a* CRISPR/Cas9 deletion and *p35S::MYB305a* over‐expressor lines in T89.
**Fig. S15** *k*‐means clustering assigns the SwAsp genotypes into more and less complex pavement cell shape clusters.
**Fig. S16** Correlation heat map of environmental variables at the original SwAsp genotype sampling sites.
**Methods S1** Propagation and transformation of *in vitro Populus* trees.
**Table S1** Primers used in this study.
Please note: Wiley is not responsible for the content or functionality of any Supporting Information supplied by the authors. Any queries (other than missing material) should be directed to the *New Phytologist* Central Office.

## Data Availability

All data supporting the findings of this study are included in the main text and Supporting Information Datasets S1 and S2, and Figs S1–S16. Raw pavement cell shape and size feature data are publicly available at doi: 10.5061/dryad.kwh70rzm9.
