# Supplementary material for: A drought stress‐induced MYB transcription factor regulates pavement cell shape in leaves of European aspen (Populus tremula)
Source: New Phytol. 2026 Jul 5;251(5):2688–705. doi: 10.1111/nph.71399 (PMC13443217; doi:10.1111/nph.71399)
Supplement: Supplementary file 1 [file NPH-251-2688-s003.pdf]

New Phytologist

***New Phytologist* Supporting Information**

Article title: **A drought stress-induced MYB transcription factor regulates pavement cell shape in leaves of European aspen (*Populus tremula*)**

Authors: Sijia Liu (刘斯佳), Siamsa M. Doyle, Kathryn M. Robinson, Zahra Rahneshan, Nathaniel R. Street, Stéphanie Robert

Article acceptance date: 14 June 2026

The following Supporting Information is available for this article:

Dataset S1 Pavement cell shape- and size-related features of Swedish Aspen (SwAsp) genotypes in the field.

Dataset S2 The top-ranked 1000 genes for each cell feature based on significance of the association of its SNPs with that feature.

Fig. S1 Pavement cell shape complexity varies among genotypes of Swedish Aspen in the field.

Fig. S2 Pavement cell features of representative SwAsp genotypes of more and less complex cell shape in the field.

Fig. S3 Pavement cell images of representative SwAsp genotypes of more and less complex cell shape in the field.

Fig. S4 Identification of a SNP in *MYB305a* associated with pavement cell shape complexity in European aspen.

Fig. S5 Broad-sense heritability and genetic correlations of the pavement cell features in European aspen.

Fig. S6 Expression of *MYB305a* in SwAsp genotypes and the closest phylogenetic relatives of *MYB305a* in European aspen and Arabidopsis.

Fig. S7 Pavement cell shape complexity varies among genotypes of juvenile European aspen in the glasshouse.

Fig. S8 Closest phylogenetic relatives of *MYB305a* in European aspen and hybrid aspen clone T89 and pavement cell shape complexity in terminal leaves of juvenile T89 in the glasshouse.

Fig. S9 Selection of genetically transformed *pMYB305a::GFP*, *MYB305a* CRISPR/Cas9 deletion and *p35S::MYB305a* over-expressor lines in T89.

Fig. S10 Shoot height in *MYB305a* CRISPR/Cas9 deletion and *p35S::MYB305a* over-expressor lines in T89.

Fig. S11 Line selection and shoot height in *MYB305a MYB305b* CRISPR/Cas9 deletion lines in T89.

Fig. S12 Pavement cell shape features in *MYB305a MYB305b* CRISPR/Cas9 deletion lines in T89.

Fig. S13 Effects of drought stress on shoot height and pavement cell shape features in T89 leaves of different developmental stages.

Fig. S14 Effects of drought stress on shoot height in *MYB305a* CRISPR/Cas9 deletion and *p35S::MYB305a* over-expressor lines in T89.

Fig. S15 *k*-means clustering assigns the SwAsp genotypes into more and less complex pavement cell shape clusters.

Fig. S16 Correlation heat map of environmental variables at the original SwAsp genotype sampling sites.

Table S1 Primers used in this study.

Methods S1 Propagation and transformation of *in vitro Populus* trees.

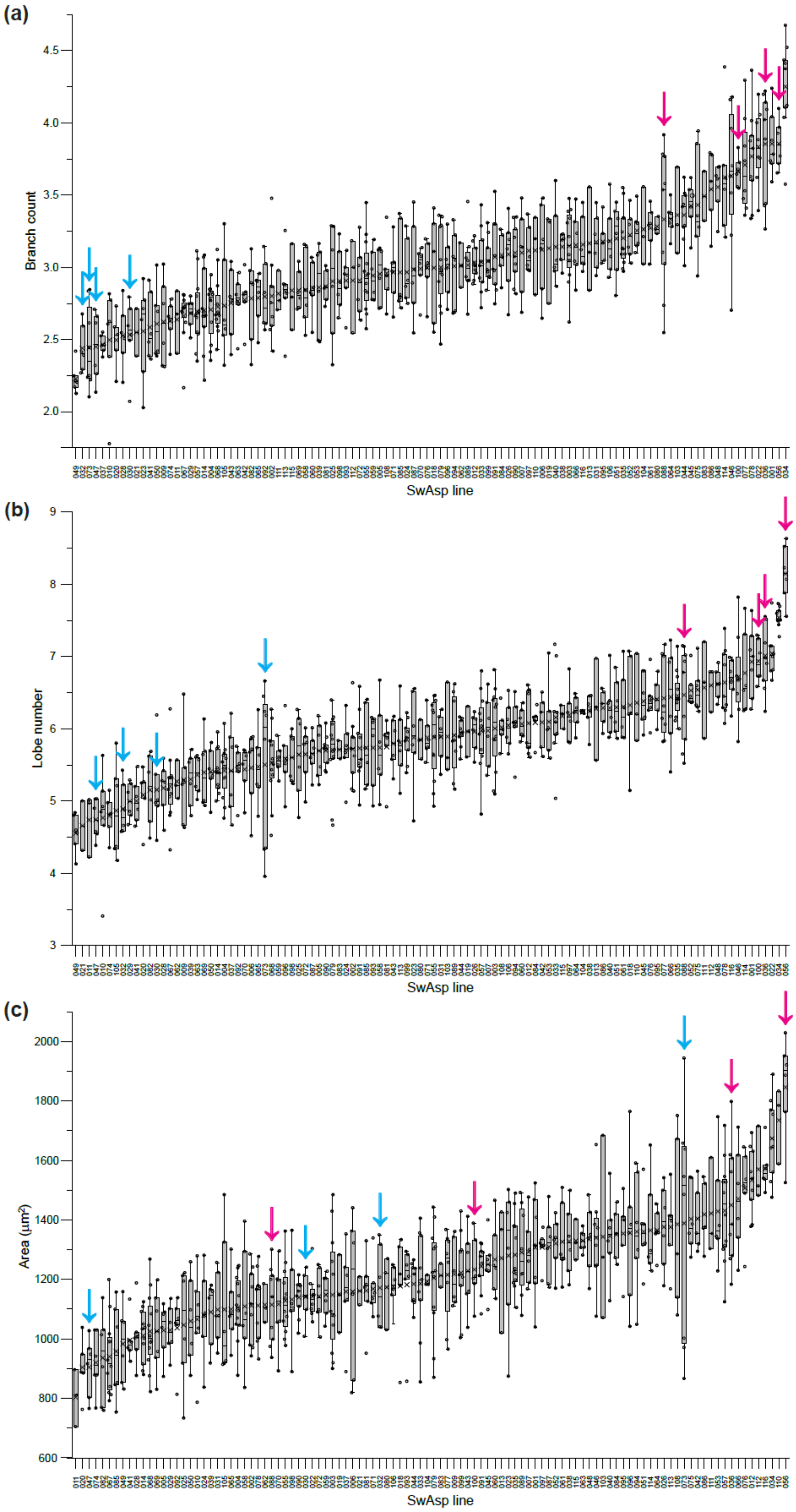
(a)
4.5
4.0
3.5
3.0
2.5
2.0
Branch count
SwAsp line
(b)
9
8
7
6
5
4
3
Lobe number
SwAsp line
(c)
2000
1800
1600
1400
1200
1000
800
600
Area (µm²)
SwAsp line

Fig. S1 Pavement cell shape complexity varies among genotypes of Swedish Aspen in the field. (a-c) Branch count (a), lobe number (b) and area (c) of pavement cells in adaxial epidermis of preformed leaf in adult trees from the SwAsp collection of European aspen (*Populus tremula*) genotypes grown in the field. To ease visualization, each data point in the box plot represents the mean value of 70-160 cells measured per leaf rather than showing each measured cell; 2-3 leaves per tree and 1-5 trees per genotype were analyzed. Those genotypes selected as representatives of more and less complex cell shape for further studies with terminal leaves of juvenile trees are indicated with magenta and cyan arrows, respectively. In the box plots, whiskers extend to the maximum/minimum values excluding outliers, a horizontal line indicates the median and x indicates the mean.

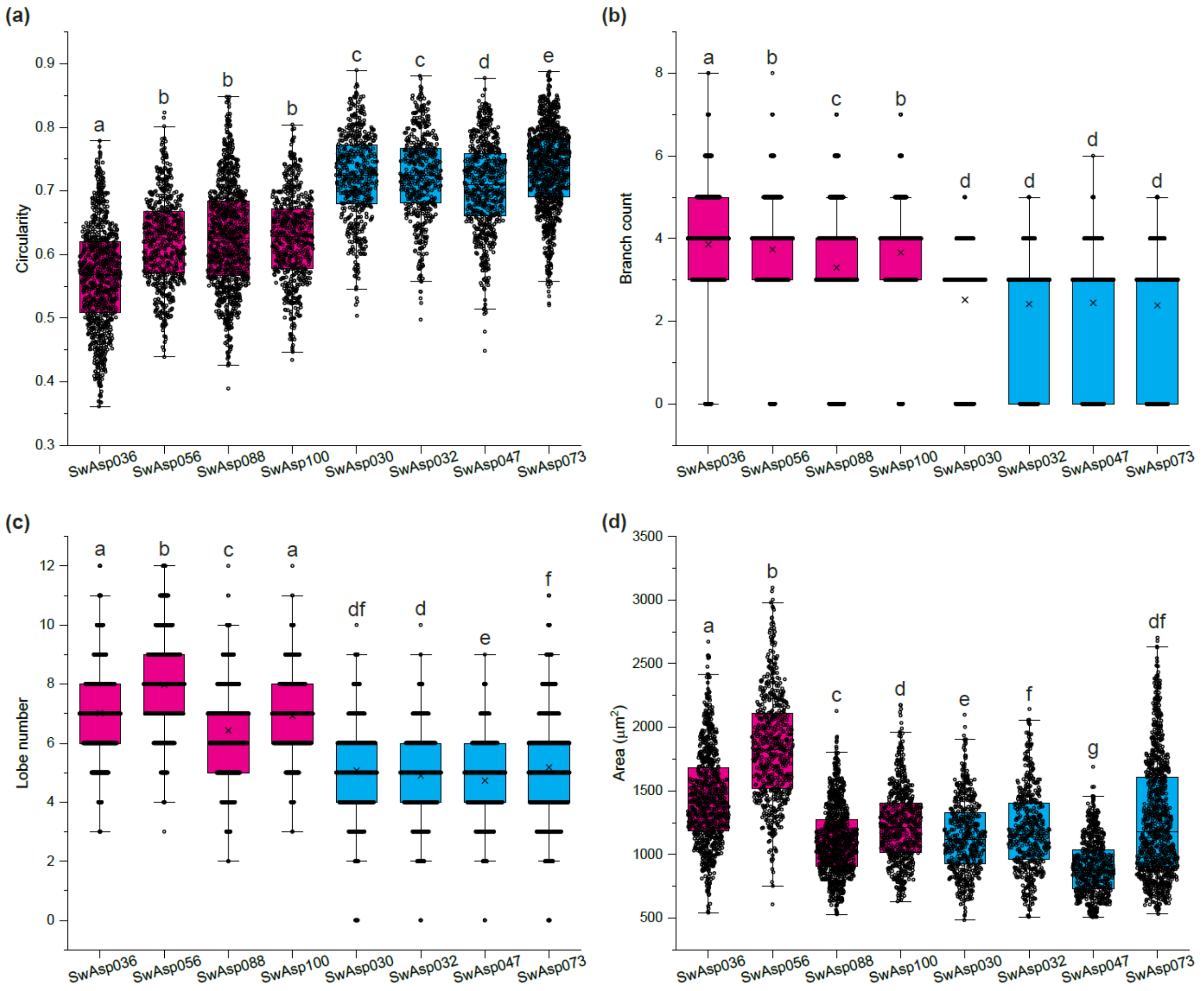

Fig. S2 Pavement cell features of representative SwAsp genotypes of more and less complex cell shape in the field. (a-d) Circularity (a), branch count (b), lobe number (c) and area (d) of pavement cells in adaxial epidermis of preformed leaf in adult trees of representative SwAsp European aspen (*Populus tremula*) genotypes of more and less complex cell shape grown in the field. Each data point in the box plot represents the value of one cell; 70-160 cells per leaf, 2-3 leaves per tree and 1-5 trees per genotype were analyzed. Different letters indicate significantly different distributions at $P < 0.05$ (Wilcoxon rank sum test). In the box plots, whiskers extend to the maximum/minimum values excluding outliers, a horizontal line indicates the median and x indicates the mean.

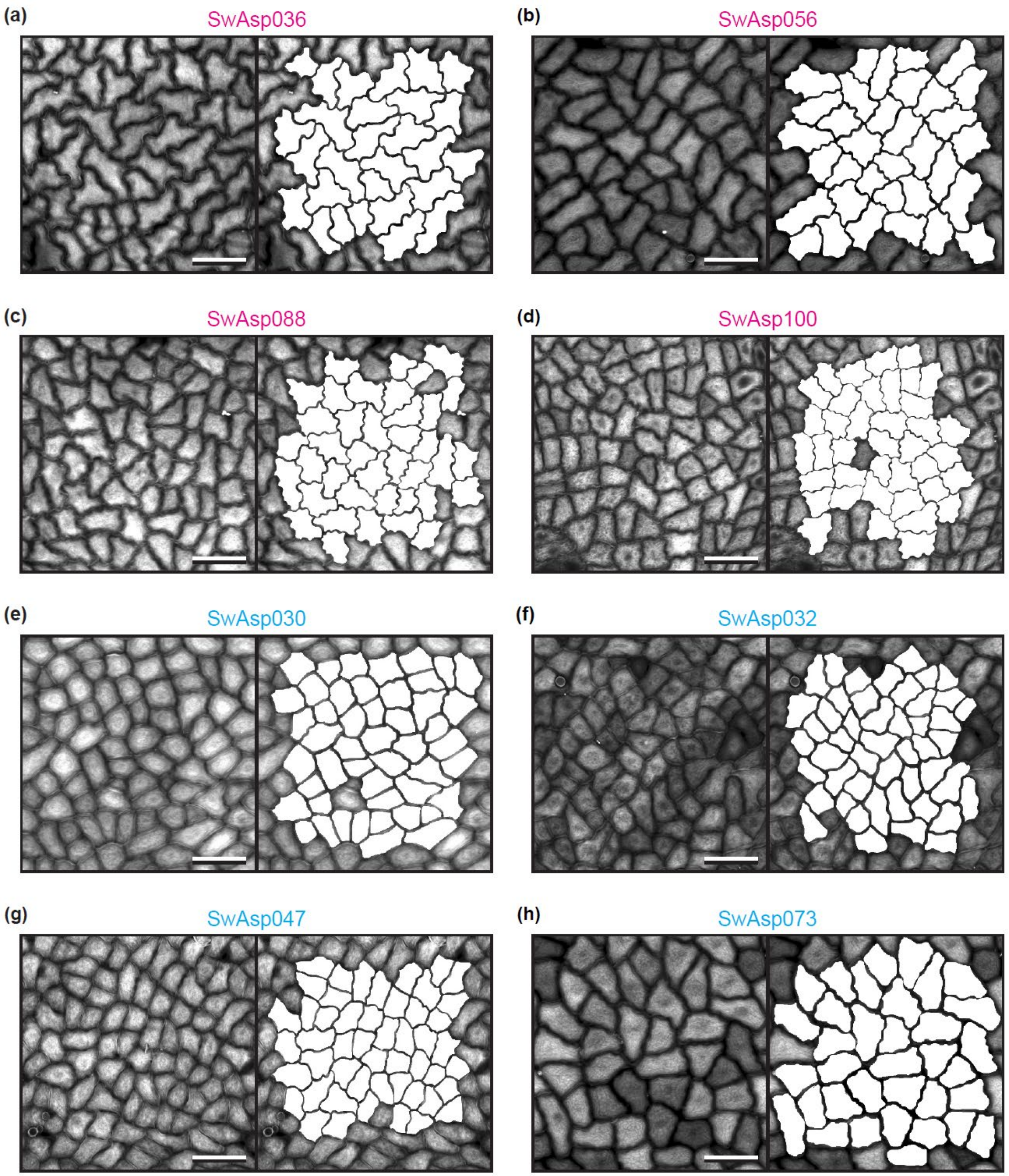

Fig. S3 Pavement cell images of representative SwAsp genotypes of more and less complex cell shape in the field. (a-h) Representative confocal microscopy images of fluorescence in calcofluor-stained pavement cells in adaxial epidermis of preformed leaf in adult trees from the SwAsp collection of European aspen genotypes grown in the field. Maximum projections of z-stacks are shown. In the right panels, free-hand cell outline drawings are overlayed onto the images shown

in the left panels. Those genotypes selected as representatives of more and less complex cell shape are shown in (a-d) [SwAsp numbers in magenta] and (e-h) [SwAsp numbers in cyan], respectively. Scale bars represent 50 µm.

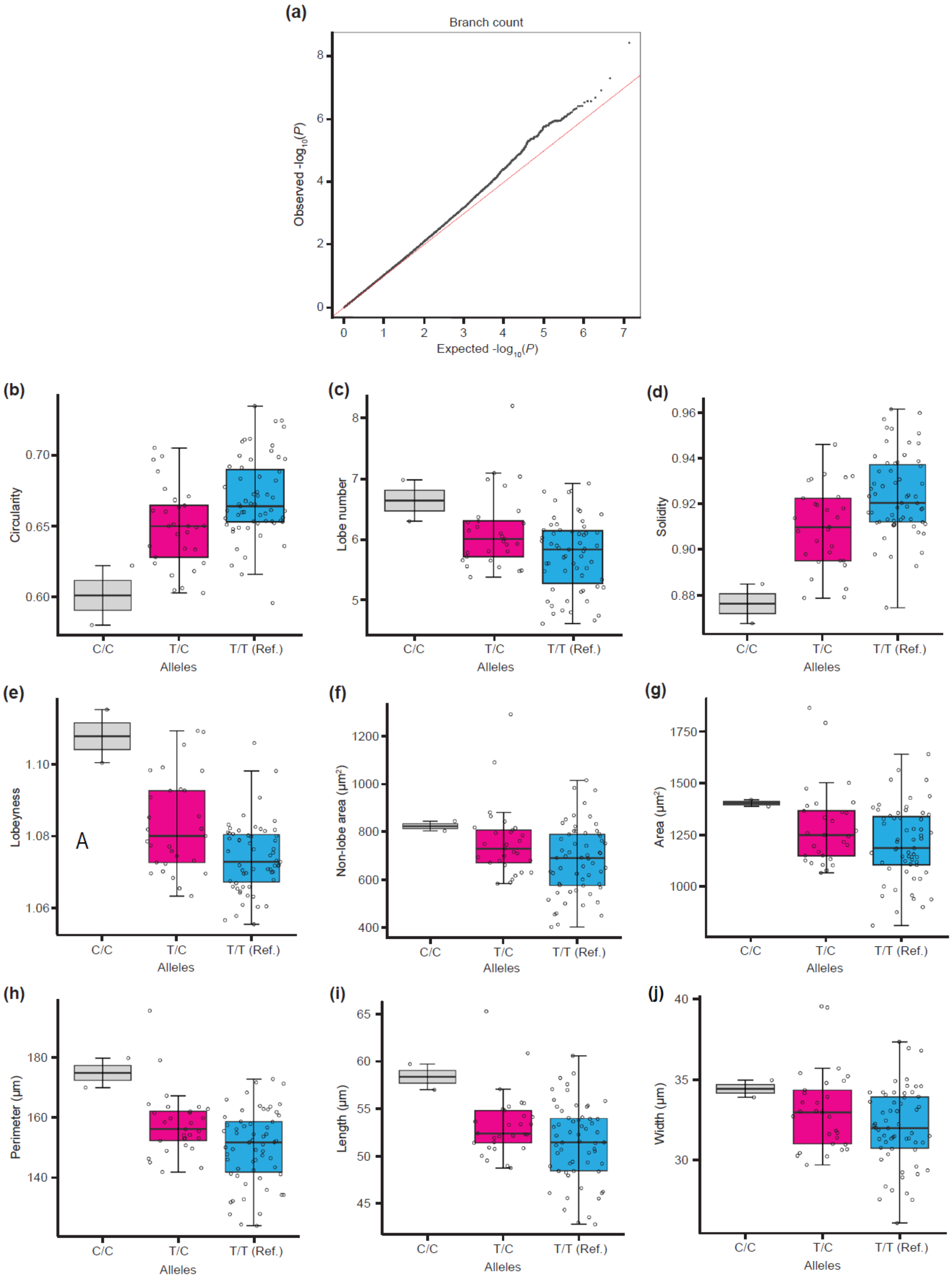
(a)
Branch count
Observed -log10(P)
Expected -log10(P)
(b)
Circularity
(c)
Lobe number
(d)
Solidity
(e)
Lobeyness
A
(f)
Non-lobe area (μm²)
(g)
Area (μm²)
(h)
Perimeter (μm)
(i)
Length (μm)
(j)
Width (μm)
C/C
T/C
T/T (Ref.)
Alleles

Fig. S4 Identification of a SNP in *MYB305a* associated with pavement cell shape complexity in European aspen. (a) Quantile-quantile (QQ) plot of the *P*-values obtained for branch count in the GWAS. (b-j) Circularity (b), lobe number (c), solidity (d), lobeyness (e), non-lobe area (f), cell area (g), perimeter (h), length (i) and width (j) of pavement cells in the SwAsp genotypes of European aspen (*Populus tremula*) analyzed, grouped according to the alleles of the candidate SNP in *MYB305a* present in the genotypes. In the box plots, whiskers extend to the maximum/minimum values excluding outliers and a horizontal line indicates the median.

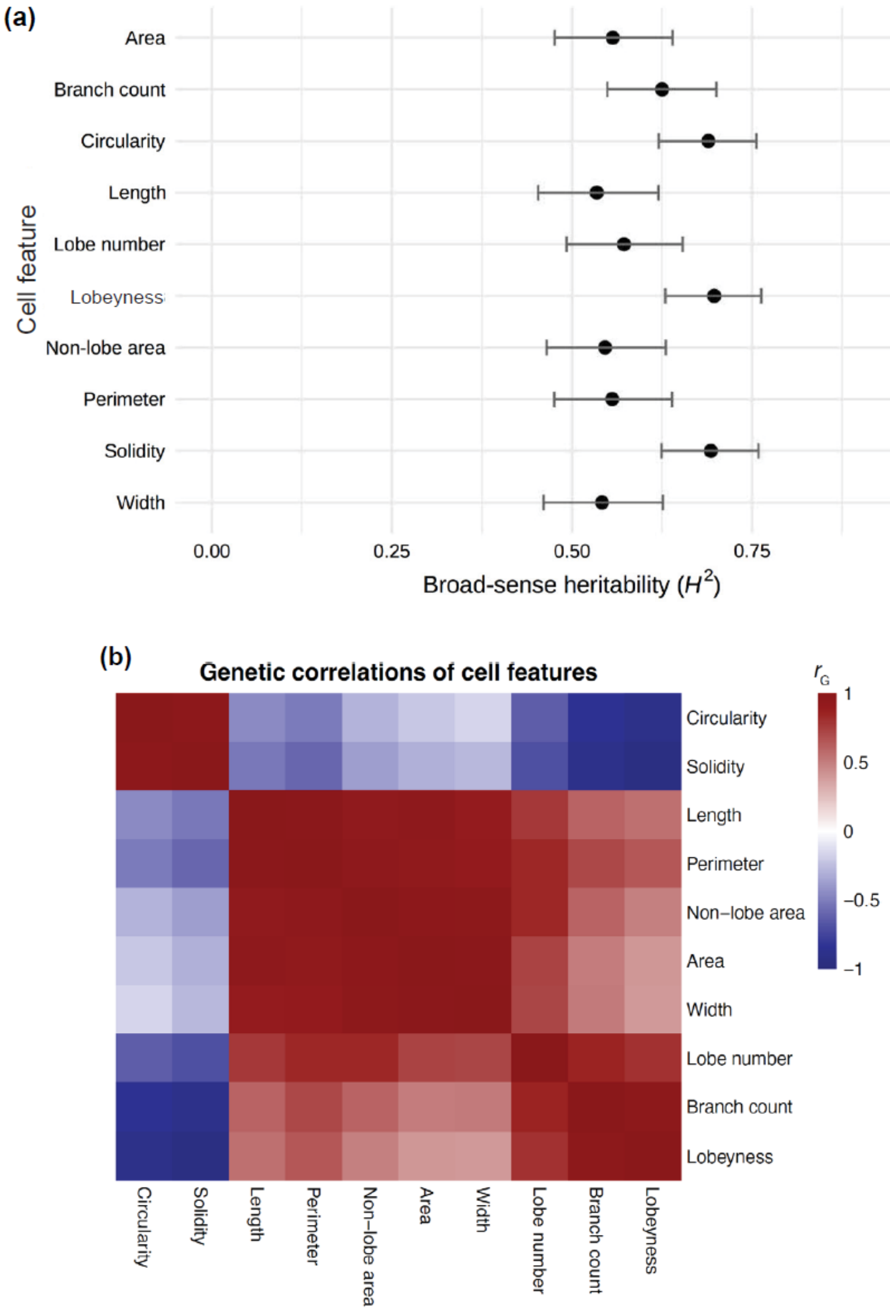

Fig. S5 Broad-sense heritability and genetic correlations of the pavement cell features in European aspen. (a-b) Broad-sense heritability, H^2 (a) and heat map of genetic correlations, r_G (b) of the shape- and size-related pavement cell features analyzed in the SwAsp genotypes of European aspen (*Populus tremula*). The whiskers in (a) show 95% confidence intervals.

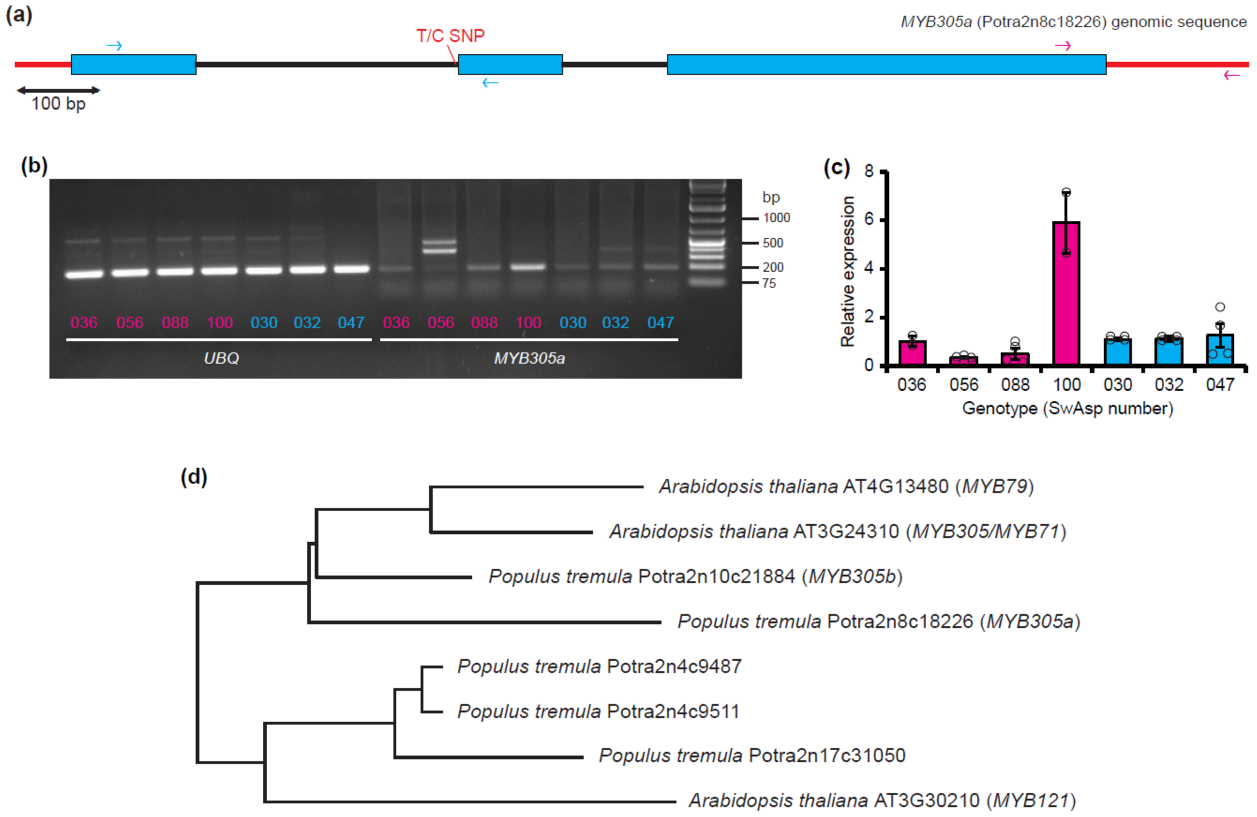

Fig. S6 Expression of *MYB305a* in SwAsp genotypes and the closest phylogenetic relatives of *MYB305a* in European aspen and Arabidopsis. (a) Genomic sequence of *MYB305a* in European aspen (*Populus tremula*) indicating the annealing sites of two primer pairs used to analyze gene expression, one of which spans the candidate T/C SNP (cyan arrows) and the other of which does not (magenta arrows). Introns are shown as black lines, UTRs as red lines and exons as cyan boxes. (b-c) Expression of *MYB305a* in terminal leaves of the representative European aspen SwAsp genotypes for more and less complex pavement cell shape (indicated in magenta and cyan, respectively), as analyzed by visualization of cDNA PCR products amplified using the primer pair spanning the candidate SNP (b), and by RT-qPCR analysis using the primer pair not spanning the candidate SNP (c). Products for the house-keeping gene *UBIQUITIN* (*UBQ*) are also shown in (b). Expression of *MYB305a* relative to that in SwAsp036 is shown in (c); error bars represent standard error of the mean. (d) Phylogenetic relationships of the closest homologs of *MYB305a* in European aspen and Arabidopsis (*Arabidopsis thaliana*).

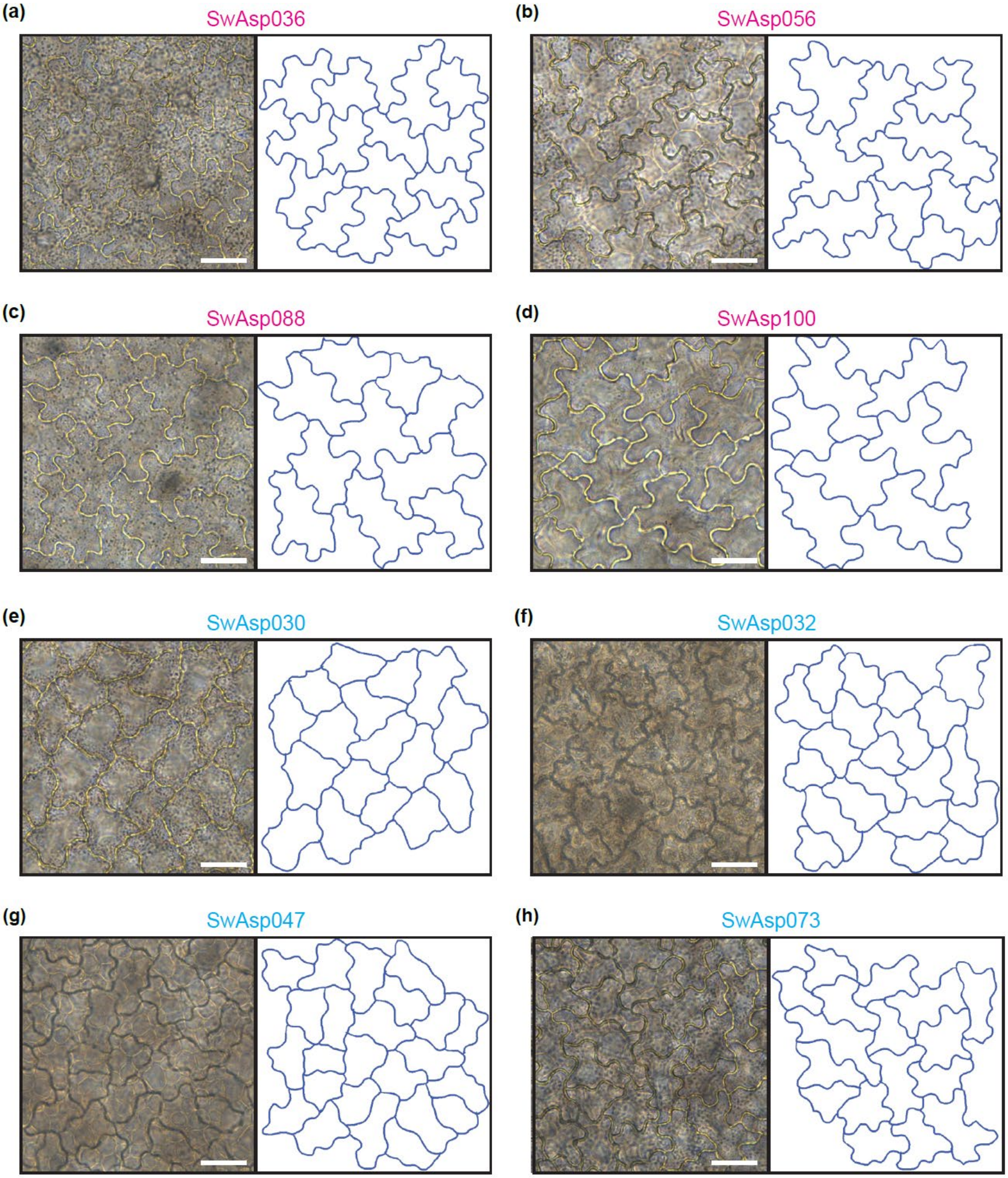

Fig. S7 Pavement cell shape complexity varies among genotypes of juvenile European aspen in the glasshouse. (a-h) Representative light microscopy images of pavement cells in adaxial epidermis of terminal leaf number 17 in juvenile trees from the SwAsp collection of European aspen (*Populus tremula*) genotypes grown in the glasshouse. In the right panels, free-hand cell outline drawings of cells in the left panel images are shown. Those genotypes selected as representatives of more and less complex cell shape are shown in (a-d) [SwAsp numbers in magenta] and (e-h) [SwAsp numbers in cyan], respectively. Scale bars represent 25 µm.

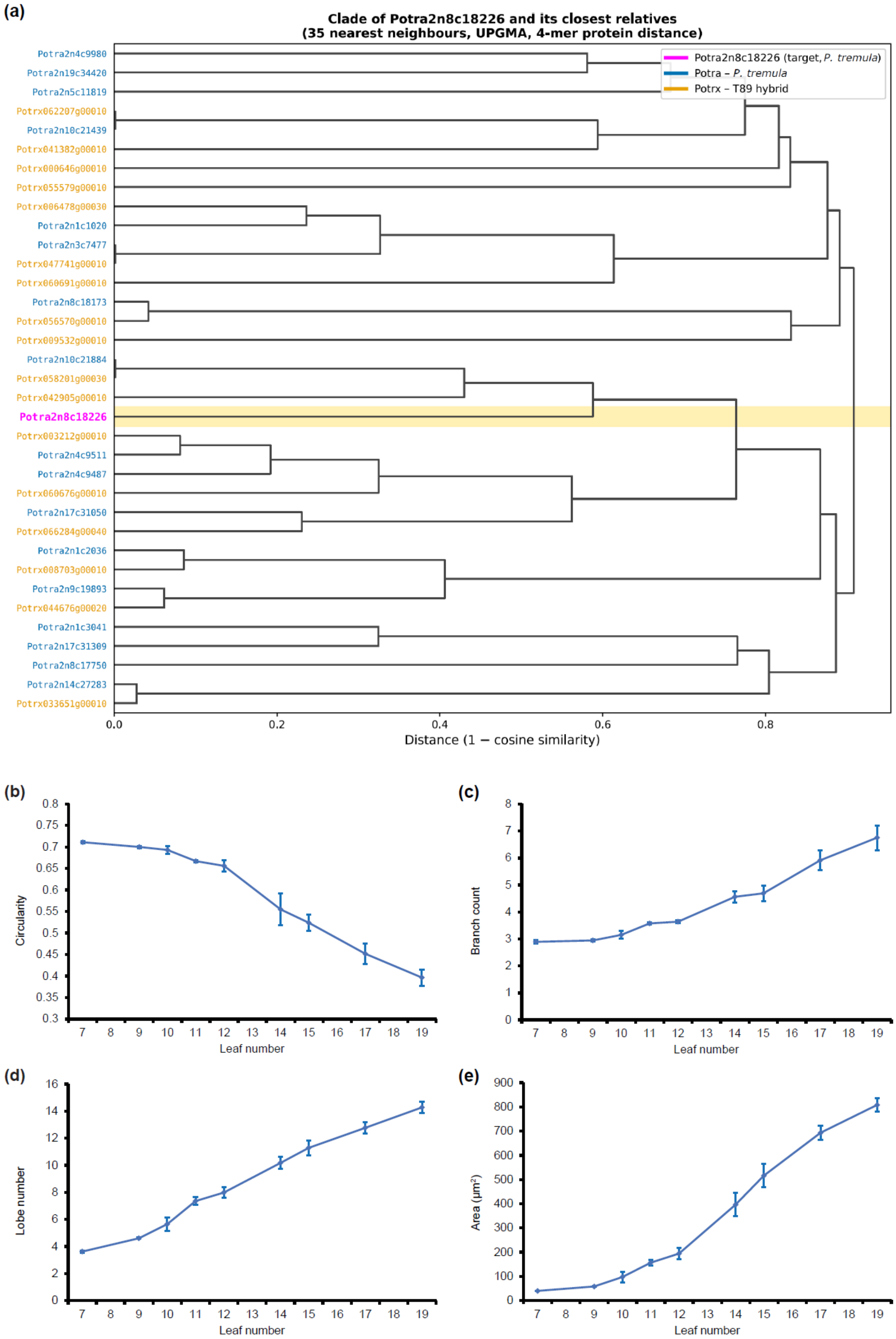

(a)
Clade of Potra2n8c18226 and its closest relatives
(35 nearest neighbours, UPGMA, 4-mer protein distance)
Potra2n8c18226 (target, P. tremula)
Potra – P. tremula
Potrx – T89 hybrid
Potra2n4c9980
Potra2n19c34420
Potra2n5c11819
Potrx062207g00010
Potra2n10c21439
Potrx041382g00010
Potrx000646g00010
Potrx055579g00010
Potrx006478g00030
Potra2n1c1020
Potra2n3c7477
Potrx047741g00010
Potrx060691g00010
Potra2n8c18173
Potrx056570g00010
Potrx009532g00010
Potra2n10c21884
Potrx058201g00030
Potrx042905g00010
Potra2n8c18226
Potrx003212g00010
Potra2n4c9511
Potra2n4c9487
Potrx060676g00010
Potra2n17c31050
Potrx066284g00040
Potra2n1c2036
Potrx008703g00010
Potra2n9c19893
Potrx044676g00020
Potra2n1c3041
Potra2n17c31309
Potra2n8c17750
Potra2n14c27283
Potrx033651g00010
Distance (1 − cosine similarity)
(b)
Circularity
Leaf number
(c)
Branch count
Leaf number
(d)
Lobe number
Leaf number
(e)
Area (μm²)
Leaf number

Fig. S8 Closest phylogenetic relatives of *MYB305a* in European aspen and hybrid aspen clone T89 and pavement cell shape complexity in terminal leaves of juvenile T89 in the glasshouse. (a) Zoomed in view of the 35 nearest neighbors of *MYB305a* (Potra2n8c18226) from a phylogenetic tree made using all 281 MYB transcription factor protein sequences in European aspen (*Populus tremula*, 148 loci [Potra]) and hybrid aspen (*Populus tremula x tremuloides*, clone T89, 133 loci [Potrx]). Pairwise distances were calculated from tetrapeptide (4-mer) amino acid composition using cosine dissimilarity, and sequences were clustered using UPGMA. The European aspen gene *MYB305a* (Potra2n8c18226, highlighted) groups in a clade with its closest T89 orthologs, including Potrx042905g00010 and Potrx058201g00030, supporting the use of the T89 hybrid as a functional model for characterising this gene. Blue: European aspen (Potra); orange: hybrid aspen (Potrx); magenta: target, *MYB305a* (Potra2n8c18226). (b-e) Circularity (b), branch count (c), lobe number (d) and area (e) of pavement cells in adaxial epidermis of terminal leaf numbers 7-19 in hybrid aspen clone T89 grown in the glasshouse. Leaf number was counted from the top down, with number 1 being the youngest leaf. Each data point represents the mean value of 89-605 cells in total, measured across 1 leaf per leaf number per tree and 3 trees. Error bars represent standard error of the mean.

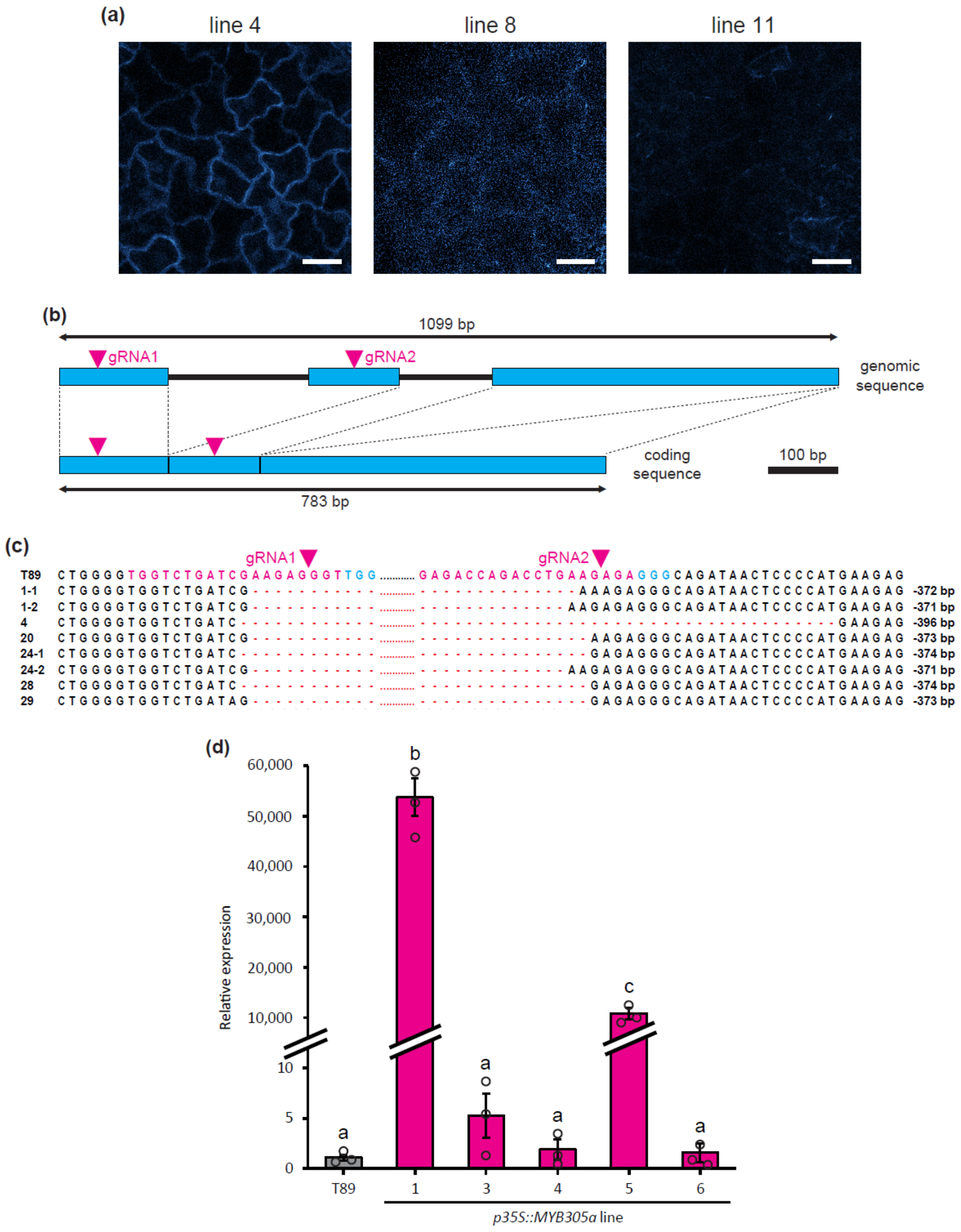
(a)
line 4
line 8
line 11
(b)
1099 bp
gRNA1
gRNA2
genomic sequence
coding sequence
100 bp
783 bp
(c)
gRNA1
gRNA2
T89 CTGGGGTGGTCTGATCGAAGAGGGTTGG GAGACCAGACCTGAAGAGAGGGCAGATAACTCCCATGAAGAG
1-1 CTGGGGTGGTCTGATCG AAAGAGGGCAGATAACTCCCATGAAGAG -372 bp
1-2 CTGGGGTGGTCTGATCG AAGAGAGGGCAGATAACTCCCATGAAGAG -371 bp
4 CTGGGGTGGTCTGATC GAAGAG -396 bp
20 CTGGGGTGGTCTGATCG AAGAGGGCAGATAACTCCCATGAAGAG -373 bp
24-1 CTGGGGTGGTCTGATC GAGAGGGCAGATAACTCCCATGAAGAG -374 bp
24-2 CTGGGGTGGTCTGATCG AAGAGAGGGCAGATAACTCCCATGAAGAG -371 bp
28 CTGGGGTGGTCTGATC GAGAGGGCAGATAACTCCCATGAAGAG -374 bp
29 CTGGGGTGGTCTGATAG GAGAGGGCAGATAACTCCCATGAAGAG -373 bp
(d)
Relative expression
60,000
50,000
40,000
30,000
20,000
10,000
10
5
0
T89
1
3
4
5
6
p35S::MYB305a line
a
b
a
a
c
a

Fig. S9 Selection of genetically transformed *pMYB305a::GFP*, *MYB305a* CRISPR/Cas9 deletion and *p35S::MYB305a* over-expressor lines in T89. (a) Representative confocal microscopy images of GFP fluorescence in pavement cells in adaxial epidermis of terminal leaf number 12 in 3 lines of *pMYB305a::GFP* in T89 (hybrid aspen, *Populus tremula* x *tremuloides*) grown in the glasshouse. Due to weak fluorescence, image acquisition settings were varied among the lines. Scale bars represent 25 µm. (b) Guide RNA (gRNA) target sites (magenta arrowheads) in the genomic and coding sequences of *MYB305a* (Potrx042905g12730) in T89. Introns are shown as black lines and exons as cyan boxes. (c) Genomic sequences of *MYB305a* in T89 and 6 *MYB305a* CRISPR/Cas9 large-fragment deletion lines (*myb305a*) at the sites of expected deletion, showing gRNA sequences (in magenta), PAM sequences (in cyan), sites of expected DNA break (magenta arrowheads) and actual deletions obtained (red dashes represent missing nucleotides). For simplicity, the genomic sequence between the gRNAs is not shown (represented as dotted lines). Two sequences are shown for those lines for which different deletions were obtained in each allele. The obtained deletion sizes are shown to the right. (d) Expression of *MYB305a* relative to that in T89 in terminal leaf number 17 in 5 *MYB305a* over-expressor (*p35S::MYB305a*, in magenta) lines grown in the glasshouse. Error bars represent standard error of the mean. Different letters indicate significantly different means at $P < 0.05$ (Tukey's HSD test).

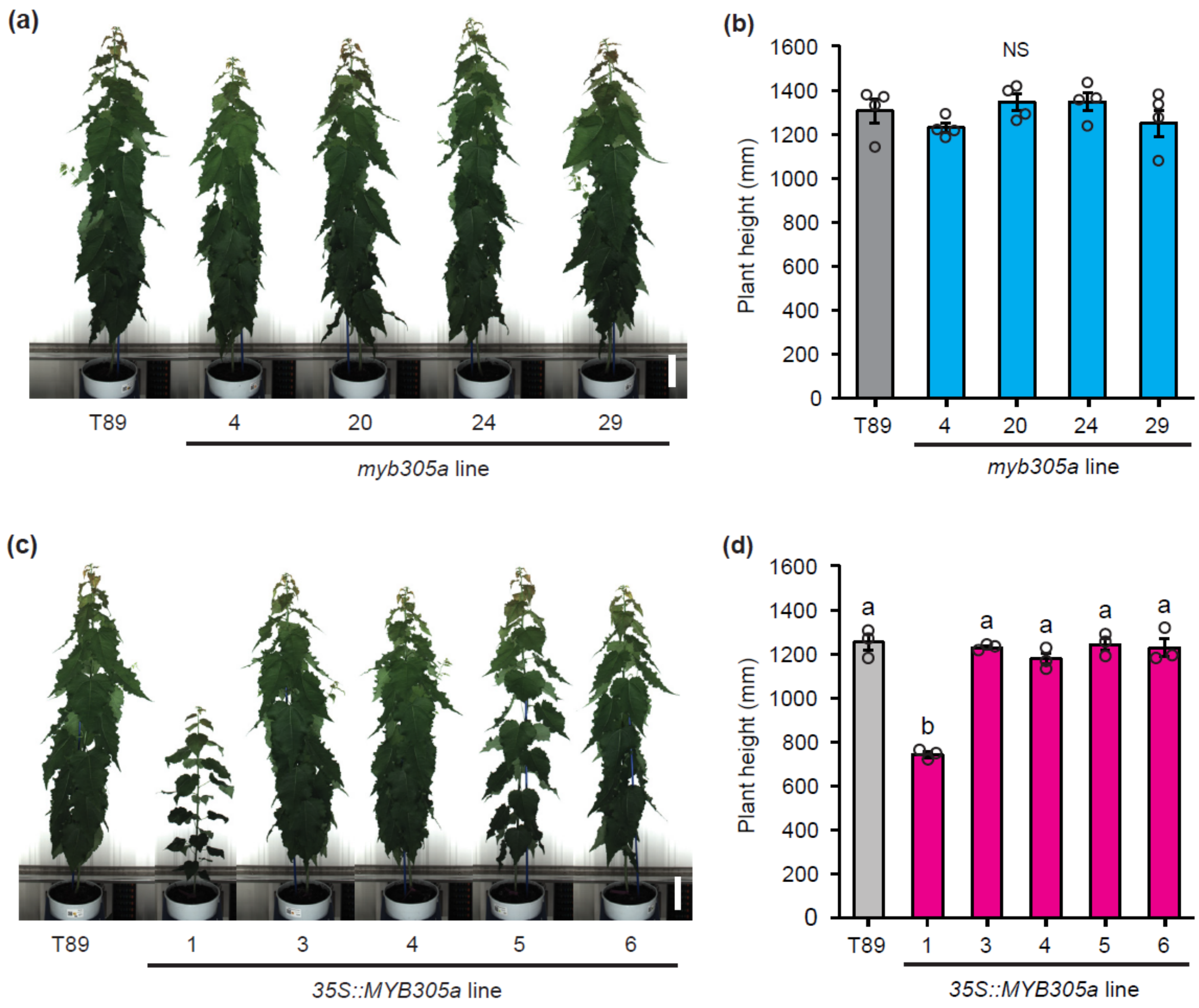

Fig. S10 Shoot height in *MYB305a* CRISPR/Cas9 deletion and *p35S::MYB305a* over-expressor lines in T89. (a-d) Shoot height in *myb305a* mutant lines (a-b) and *p35S::MYB305a* over-expressor lines (c-d) in T89 compared to T89 (hybrid aspen, *Populus tremula* x *tremuloides*) after 8 weeks of growth in soil. Images of representative plants are shown; side shoots were removed 3 and 6 weeks after potting in soil; scale bars represent 30 cm. Error bars represent standard error of the mean. Different letters indicate significantly different means at $P < 0.05$ (Tukey's HSD test; NS, not significant).

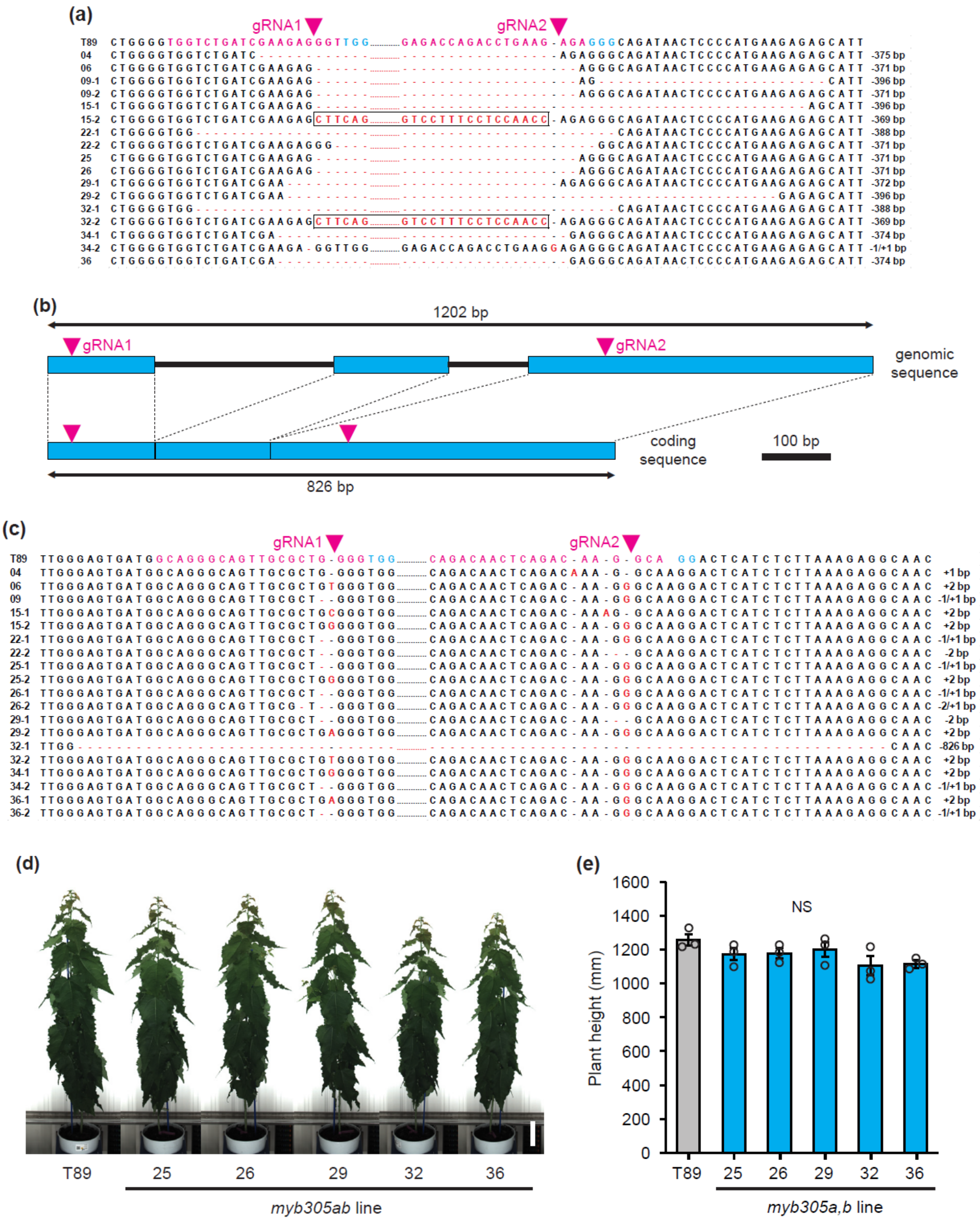

(a)
gRNA1
gRNA2
(b)
1202 bp
gRNA1
gRNA2
genomic sequence
coding sequence
100 bp
826 bp
(c)
gRNA1
gRNA2
(d)
T89
25
26
29
32
36
myb305ab line
(e)
NS
Plant height (mm)
1600
1400
1200
1000
800
600
400
200
0
T89
25
26
29
32
36
myb305a,b line

Fig. S11 Line selection and shoot height in *MYB305a MYB305b* CRISPR/Cas9 deletion lines in T89. (a) Genomic sequences of *MYB305a* in T89 and 11 *MYB305a MYB305b* CRISPR/Cas9 mutant (*myb305ab*) lines in T89 (hybrid aspen, *Populus tremula* x *tremuloides*) at the sites of expected deletion, showing guide RNA (gRNA) sequences (in magenta), PAM sequences (in cyan), sites of expected DNA break (magenta arrowheads) and actual deletions (red dashes represent missing nucleotides) or insertions (in red) obtained. An inversion was discovered in two of the lines (black boxes). For simplicity, the genomic sequence between the gRNAs is not shown (represented as dotted lines). Two sequences are shown for those lines for which different deletions were obtained in each allele. The obtained deletion/insertion sizes are shown to the right. (b) gRNA target sites (magenta arrowheads) in the genomic and coding sequences of *MYB305b* (Potrx058201g19640) in T89. Introns are shown as black lines and exons as cyan boxes. (c) Genomic sequences of *MYB305b* in T89 and the same 11 *myb305ab* lines for which *MYB305a* is shown in (a). (d-e) Shoot height in *myb305ab* mutant lines in T89 compared to T89 after 8 weeks of growth in soil. Side shoots were removed 3 and 6 weeks after potting in soil; scale bars represent 30 cm. Error bars represent standard error of the mean. NS, not significantly different means at $P < 0.05$ (Tukey's HSD test).

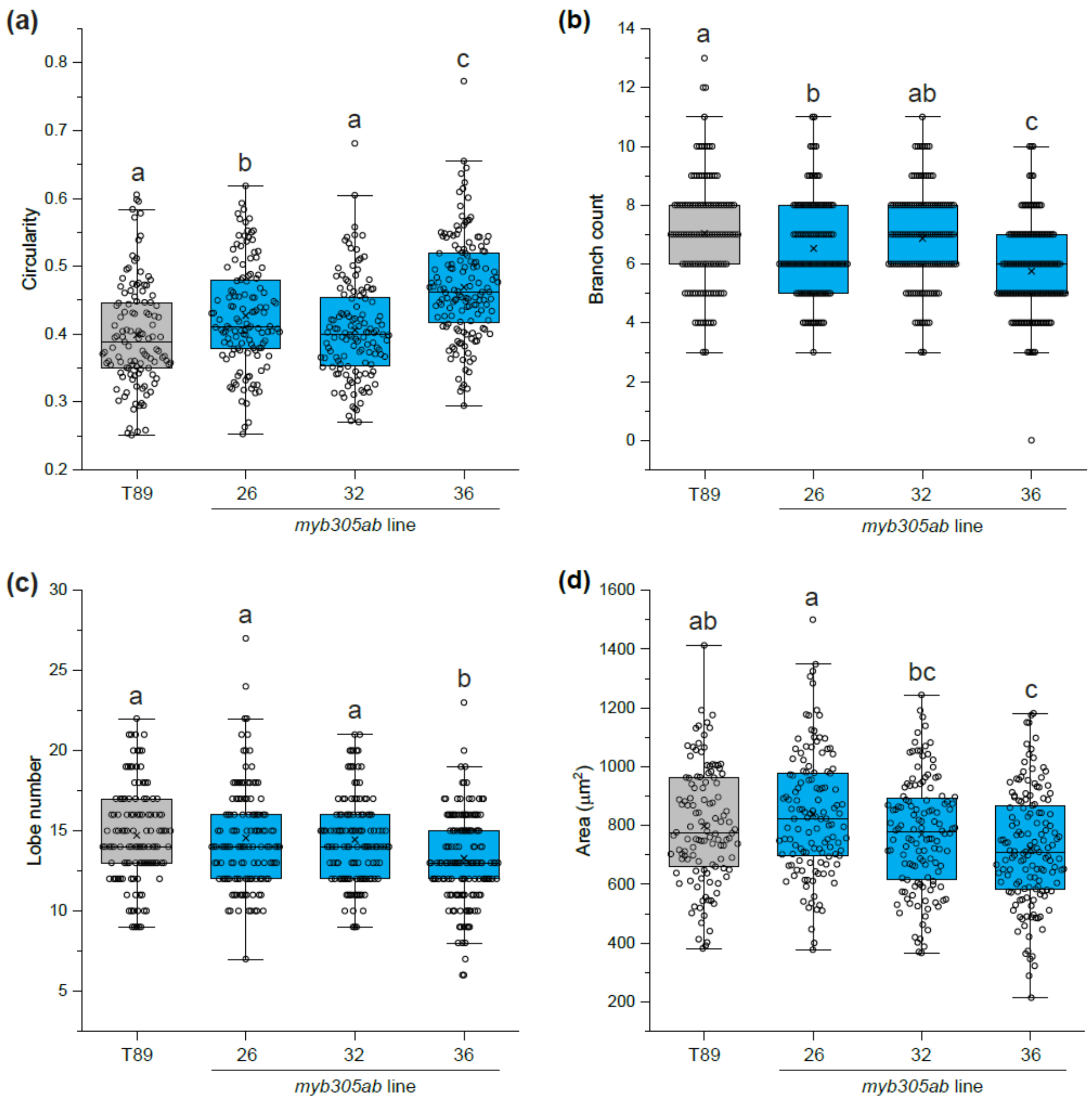

Fig. S12 Pavement cell shape features in *MYB305a MYB305b* CRISPR/Cas9 deletion lines in T89. (a-d) Circularity (a), branch count (b), lobe number (c) and area (d) of pavement cells in adaxial epidermis of terminal leaf number 17 in double *MYB305a MYB305b* CRISPR mutant (*myb305ab*, in cyan) lines in T89 (hybrid aspen, *Populus tremula* x *tremuloides*) grown in the glasshouse. Each data point in the box plot represents one of 118-145 cells in total measured across 1 leaf per tree and 3 trees per genotype. Different letters indicate significantly different distributions or means at $P < 0.05$ (Wilcoxon rank sum test for [a-c]; Tukey's HSD test for [d]). In the box plots, whiskers extend to the maximum/minimum values excluding outliers, a horizontal line indicates the median and x indicates the mean.

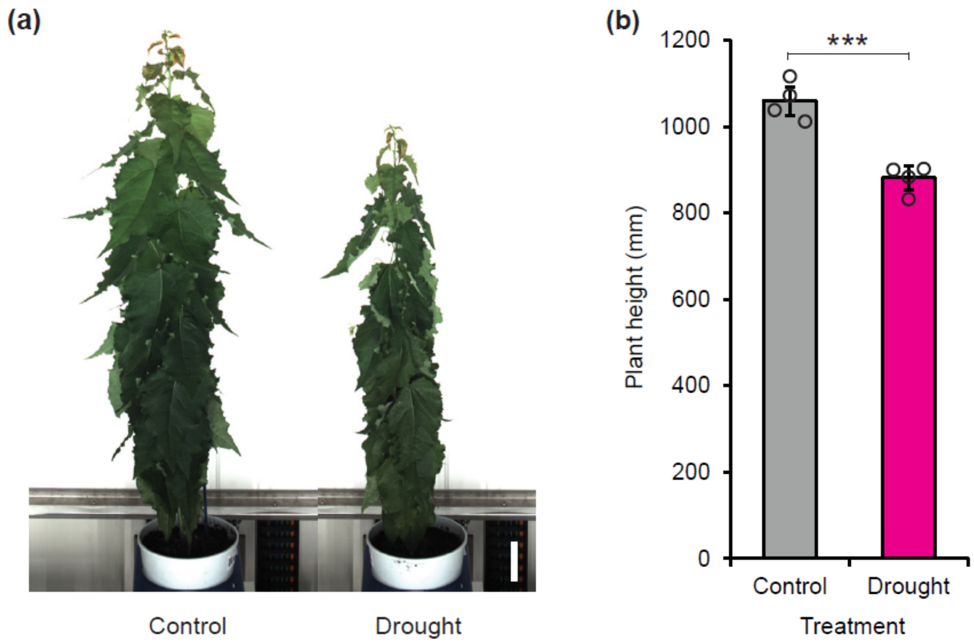

Fig. S13 Effects of drought stress on shoot height and pavement cell shape features in T89 leaves of different developmental stages. (a-b) Representative images (a) and shoot height (b) of *pMYB305a::GFP* Line 4 in T89 (hybrid aspen, *Populus tremula* x *tremuloides*) in control and drought conditions at time of leaf sampling for pavement cell imaging (after 7 weeks of growth in soil). Side shoots were removed 3 and 6 weeks after potting in soil. Scale bar represents 20 cm. Error bars represent standard error of the mean. Asterisks indicate significantly different means between the control and drought treatment according to the Student's t-test (***P < 0.001).

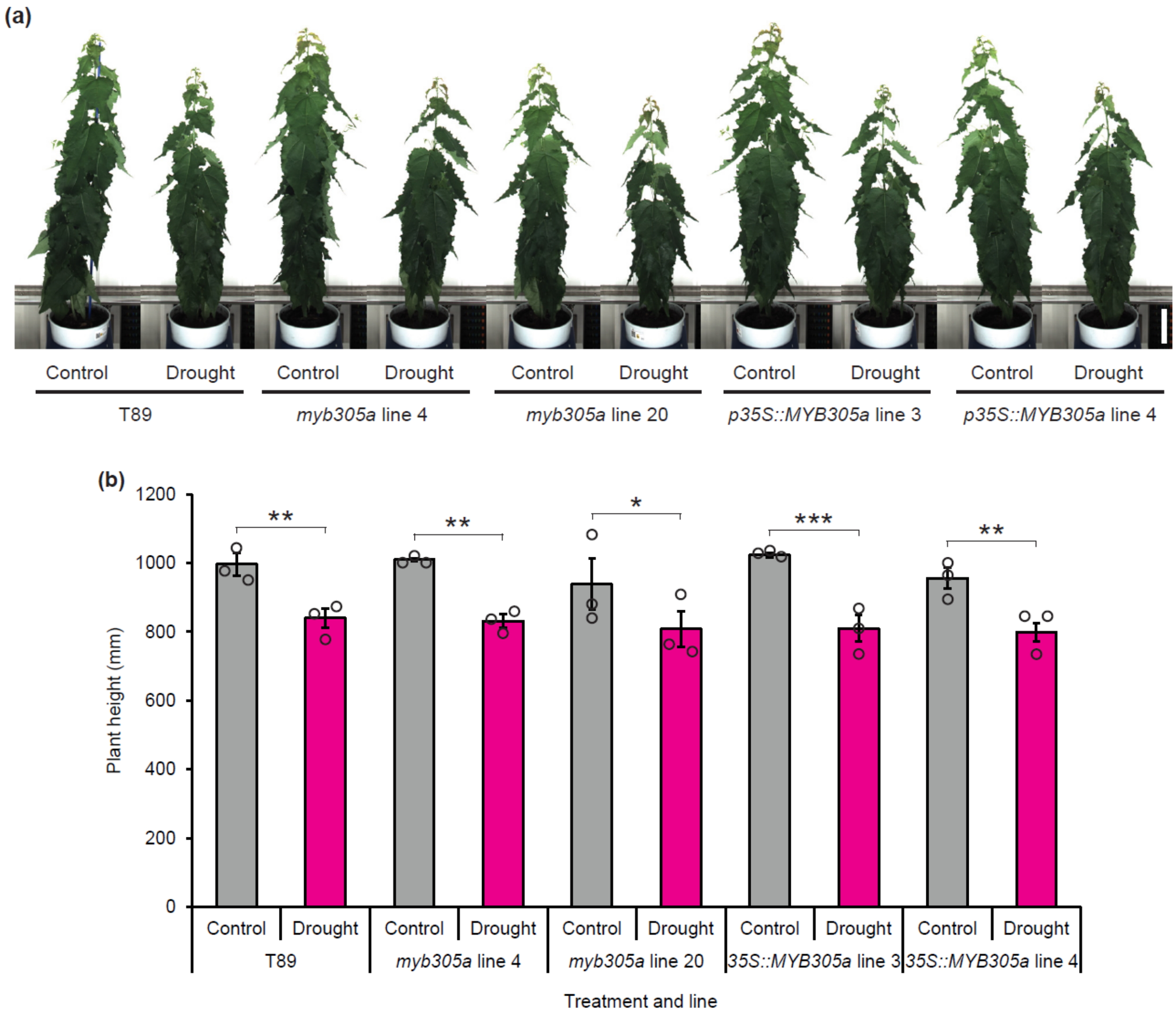

Fig. S14 Effects of drought stress on shoot height in *MYB305a* CRISPR/Cas9 deletion and *p35S::MYB305a* over-expressor lines in T89. (a-b) Representative images (a) and shoot height (b) of T89 (hybrid aspen, *Populus tremula* x *tremuloides*), *myb305a* mutant lines in T89 and *p35S::MYB305a* over-expressor lines in T89 in control and drought conditions at time of leaf sampling for pavement cell imaging (after 7 weeks of growth in soil). Side shoots were removed 3 and 6 weeks after potting in soil. Scale bars represent 30 cm. Error bars represent standard error of the mean. Asterisks indicate significantly different means between the control and drought treatment according to the Student's t-test (*$P < 0.05$; **$P < 0.01$; ***$P < 0.001$).

(a)

SwAsp no.	Cluster	SwAsp no.	Cluster	SwAsp no.	Cluster	SwAsp no.	Cluster
001	2	034	2	061	2	088	2
002	1	035	2	062	1	089	2
003	2	036	2	063	1	090	2
004	1	037	1	064	2	091	2
005	1	038	2	065	1	092	1
006	2	039	1	066	2	093	1
007	2	040	2	067	1	094	2
009	1	041	1	068	1	095	2
011	1	042	2	069	1	096	2
012	2	043	1	070	1	097	2
013	2	044	2	071	1	098	1
014	1	045	2	072	1	099	2
018	1	046	2	073	1	100	2
019	2	047	1	074	1	103	2
020	1	048	2	075	2	104	2
022	2	049	1	076	2	105	1
023	1	050	1	077	2	106	2
024	1	051	2	078	2	108	2
025	1	052	2	079	1	110	2
026	2	053	2	080	2	111	2
028	1	055	1	081	1	112	2
029	1	056	2	082	1	113	2
030	1	057	2	084	2	114	2
031	1	058	1	085	1	115	2
032	1	059	1	086	2	116	2
033	2	060	2	087	2		

(b)

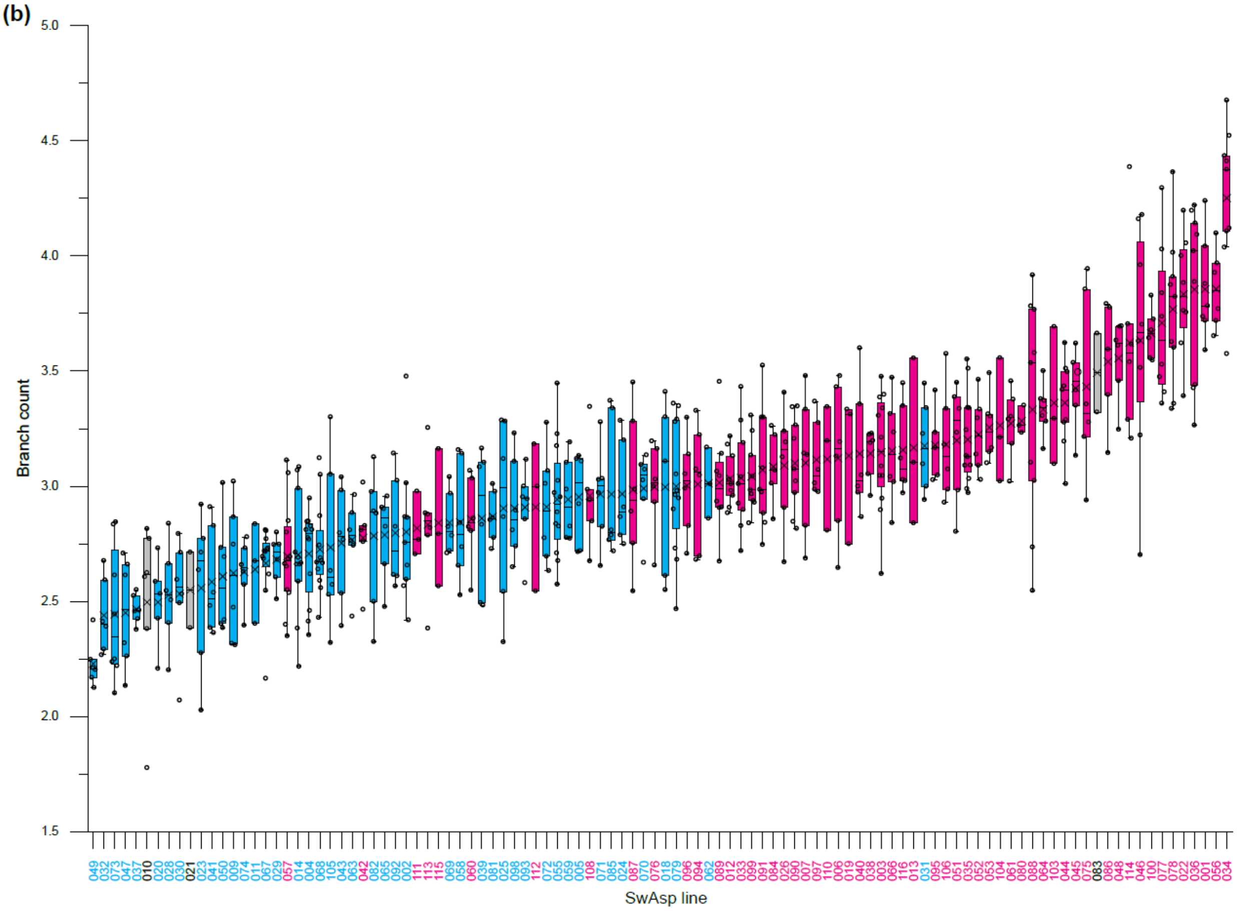

Fig. S15 *k*-means clustering assigns the SwAsp genotypes into more and less complex pavement cell shape clusters. (a) *k*-means cluster analysis of the SwAsp genotypes of European aspen (*Populus tremula*) based on all cell shape and size features. The representative genotypes for more and less complex pavement cell shape are indicated in magenta and cyan, respectively. (b) Branch count of pavement cells in the SwAsp genotypes, as shown in Fig. S1a, but color-coded according to the cluster to which that genotype was assigned by *k*-means cluster analysis, with cyan used for cluster 1 and magenta for cluster 2. In the box plot, whiskers extend to the maximum/minimum values excluding outliers, a horizontal line indicates the median and x indicates the mean.

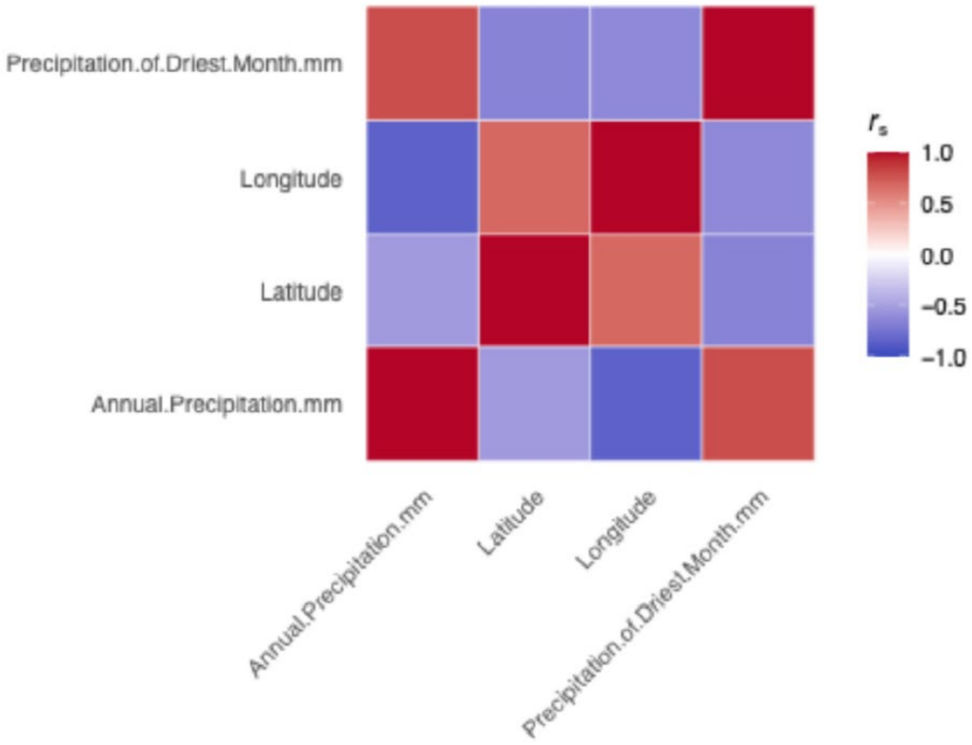

Fig. S16 Correlation heat map of environmental variables at the original SwAsp genotype sampling sites. Relationship, between latitude, longitude and average precipitation metrics for the original sampling sites of the SwAsp genotypes of European aspen (*Populus tremula*) visualized with a Spearman rank correlation (r_S) matrix.

Primer name	Primer sequence (5’ to 3’)
proPtMYB305a_F_attB1	GGGGACAAGTTTGTACAAAAAAGCAGGCTTCGAAGGAGATAGAACCATGAATGGCGAATGGCAAATAAC
proPtMYB305a_ R_attB2	GGGGACCACTTTGTACAAGAAAGCTGGGTGGTATAAAGGGAGGAATTAAGGAAAAG
PtMYB305a_CDS_F_attB1	GGGGACAAGTTTGTACAAAAAAGCAGGCTTCGAAGGAGATAGAACCATGTCTTGGGGAGTGATGG
PtMYB305a_CDS_R_attB2	GGGGACCACTTTGTACAAGAAAGCTGGGTGACAAAAAGGTGCCACTA
PtMYB305a_sgRNA1_T1F	ATATATGGTCTCGATTGTGGTCTGATCGAAGAGGGTGTTTTAGAGCTAGAAATAG
PtMYB305a_sgRNA2_T2R	ATTATTGGTCTCTAAACTCTCTTCAGGTCTGGTCTCCAATCTCTTAGTCGACTCTAC
PtMYB305a_sgRNA2_T2F	ATATTATTGGTCTCAAGATTGGAGACCAGACCTGAAGAGAGTTTTAGAGCTAGAAATAG
PtMYB305b_sgRNA1_T3F	ATATTATTGGTCTCAGTGATTGCAGGGCAGTTGCGCTGGGGGTTTTAGAGCTAGAAATAG
PtMYB305b_sgRNA3_T4R	ATTATTGGTCTCTAAACTGCCTTGTCTGAGTTGTCTCAATCACTACTTCGACTCTAG
PttMYB305a_2sgR_gF	CCCCCATATGCACAGAATTT
PttMYB305a_2sgR_gR	CAGTAAGGATGGTGGGAGTTC
PttMYB305b_4sgR_gF	AAGACAGCCTTTGCCAACAC
PttMYB305b_4sgR_gR	TTGCTGTTGTTGCTGCTGTA
PttMYB305a_RT-qPCR_F1	TCTGATCGAAGAGGGTTGGA
PttMYB305a_RT-qPCR_R1	GCCCTCTCTTCAGGTCTGGT
PttMYB305a_RT-qPCR_F2	TGGCAATCTATGTGCAACAA
PttMYB305a_RT-qPCR_R2	TTTGCGTTATCAAGGTTCCA
PtUBQ_RT-qPCR_F	GTTGATTTTTGCTGGGAAGC
PtUBQ_RT-qPCR_R	GATCTTGGCCTTCACGTTGT

Table S1 Primers used in this study. Names and sequences of all primers used. Pt refers to European aspen (*Populus tremula*), Ptt refers to hybrid aspen (*Populus tremula* x *tremuloides*), pro refers to promoter region, CDS refers to coding sequence and sgRNA or sgR refers to single guide RNA.

Methods S1 Propagation and transformation of *in vitro Populus* trees.

Trees of Swedish aspen (SwAsp) genotypes of European aspen (*Populus tremula*) and hybrid aspen (*Populus tremula* x *tremuloides*) clone T89 were obtained from the *in vitro* collections of *Populus* species maintained via shoot cutting propagation by the Umeå Plant Science Centre (UPSC) Poplar Transgenics Facility. The *in vitro* plants were maintained in sterile conditions in half-strength Murashige and Skoog (MS) medium with 3.5 g l^{-1} gelrite at pH 5.6 and incubated in 22°C under an 18/6 h light/dark regime. White paper was used as shade for the first 2 weeks of growth of new cuttings. All transgenic lines were created in the T89 background and also maintained *in vitro*. The transformation constructs were introduced into *Agrobacterium tumefaciens* strain GV3101 by electroporation, followed by growth in liquid culture with lysogeny broth (LB) medium at 28°C with constant rotation. Antibiotic concentrations used for *Agrobacterium* selection were 20 µg ml^{-1} rifampicin, 30 µg ml^{-1} gentamicin, 10 µg ml^{-1} tetracyclin and the relevant bacterial resistance antibiotic (100 µg ml^{-1} spectinomycin or 50 µg ml^{-1} kanamycin). *Agrobacterium* pellets were obtained once the culture OD at 600 nm reached 0.3-0.8 and transformation was then performed on T89 by the UPSC Poplar Transgenics Facility. The pellets were resuspended in full-strength MS medium at pH 5.8 supplemented with 20 µM acetosyringone, to which 100-200 sterile stem and petiole pieces of 5-10 mm in length were added in petri dishes and incubated for 1 hour. All subsequent steps were performed in sterile conditions. The plant pieces were transferred to plates of MS1 medium (full-strength MS medium with 2% sucrose, 0.07% 4-morpholineethanesulfonic acid (MES), 0.2 µg ml^{-1} 6-benzylaminopurine (BAP), 0.1 µg ml^{-1} Indole-3-butyric acid (IBA), 0.01 µg ml^{-1} thidiazuron (TDZ) and 0.6% plant agar) at pH 5.8 and incubated in darkness for 2 days at 22°C. The explants were then washed twice with 500 µg ml^{-1} cefotaxime before transferring to plates of MS1 medium at pH 5.6 supplemented with 500 µg ml^{-1} cefotaxime and the relevant plant resistance antibiotic (25 µg ml^{-1} hygromycin or 80 µg ml^{-1} kanamycin), which were incubated in *in vitro* growth conditions. The explants were transferred to fresh plates every 2 weeks until calli were formed, after which one callus per explant was removed and transferred to fresh plates every 2 weeks until reaching 1-2 cm in diameter. At this point, calli were transferred to MS2 medium (MS1 without MES or TDZ, with 3.5 g l^{-1} gelrite instead of agar) at pH 5.6 supplemented with 500 µg ml^{-1} cefotaxime and incubated in *in vitro* conditions until shoots were formed. Finally, shoot cuttings were transferred to *in vitro* growth medium and maintained in *in vitro* conditions.
